# Materials Genes of CO_2_ Hydrogenation on Supported Cobalt Catalysts: An Artificial Intelligence Approach Integrating Theoretical and Experimental Data

**DOI:** 10.1021/jacs.3c12984

**Published:** 2024-02-20

**Authors:** Ray Miyazaki, Kendra S Belthle, Harun Tüysüz, Lucas Foppa, Matthias Scheffler

**Affiliations:** † The NOMAD Laboratory at the Fritz-Haber-Institut of the Max-Planck-Gesellschaft and IRIS-Adlershof of the Humboldt-Universität zu Berlin, Faradayweg 4-6, Berlin 14195, Germany; ‡ Max-Planck-Institut für Kohlenforschung, Kaiser-Wilhelm-Platz 1, Mülheim an der Ruhr 45470, Germany

## Abstract

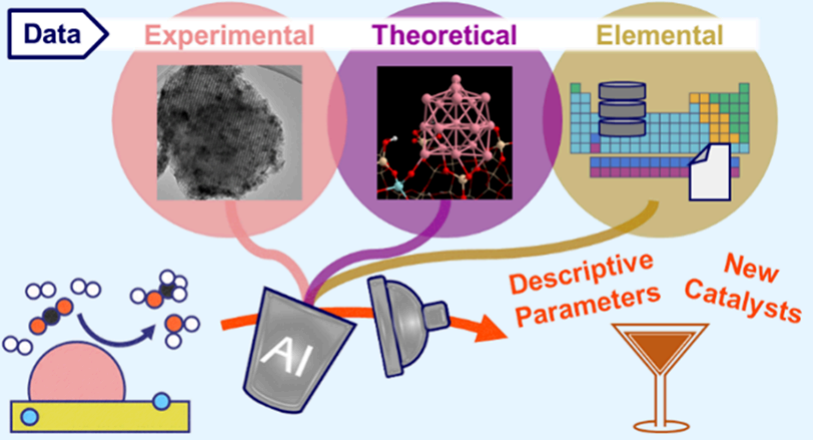

Designing materials for catalysis is challenging because the performance is governed by an intricate interplay of various multiscale phenomena, such as the chemical reactions on surfaces and the materials’ restructuring during the catalytic process. In the case of supported catalysts, the role of the support material can be also crucial. Here, we address this intricacy challenge by a symbolic-regression artificial intelligence (AI) approach. We identify the key physicochemical parameters correlated with the measured performance, out of many offered candidate parameters characterizing the materials, reaction environment, and possibly relevant underlying phenomena. Importantly, these parameters are obtained by both experiments and ab initio simulations. The identified key parameters might be called “materials genes”, in analogy to genes in biology: they correlate with the property or function of interest, but the explicit physical relationship is not (necessarily) known. To demonstrate the approach, we investigate the CO_2_ hydrogenation catalyzed by cobalt nanoparticles supported on silica. Crucially, the silica support is modified with the additive metals magnesium, calcium, titanium, aluminum, or zirconium, which results in six materials with significantly different performances. These systems mimic hydrothermal vents, which might have produced the first organic molecules on Earth. The key parameters correlated with the CH_3_OH selectivity reflect the reducibility of cobalt species, the adsorption strength of reaction intermediates, and the chemical nature of the additive metal. By using an AI model trained on basic elemental properties of the additive metals (e.g., ionization potential) as physicochemical parameters, new additives are suggested. The predicted CH_3_OH selectivity of cobalt catalysts supported on silica modified with vanadium and zinc is confirmed by new experiments.

## 1 Introduction

Heterogeneous catalysis is one of the essential technologies in modern societies, since it has been utilized for decomposing toxic species, generating valuable chemicals, and for many more industrial applications.^1−6^ Thus, there is a great demand for discovering and designing new catalytic materials that show higher performance than the available ones. Furthermore, catalysis is not only relevant to industrial applications but also links to fundamental science. For instance, organic molecules are generated by CO_2_ hydrogenation at hydrothermal vents, which are fissures on the seafloor that discharge heated water.^10,11^ This reaction is catalyzed by metal-containing materials. Because hydrothermal vents existed at the early Earth, they are considered as one of the candidate systems that produced the first organic molecules, eventually enabling the emergence of life.

Identifying key physicochemical parameters that describe the catalytic performance is a key step to design new catalytic materials and to understand the underlying phenomena. However, heterogeneous catalysis is governed by a complex and intricate interplay of several multiscale processes, such as transport of reactants, products, and heat in reactors, dynamical phase and structural transitions of catalytic materials during reactions, and chemical reactions on catalyst surfaces.^1−3,12^ The time scales of those processes are also different. In particular, the catalytic material is often deposited on a support, for instance, metal nanoparticles are commonly supported on oxides. In these systems, the support material can also play a critical role in catalysis.^4,7−9^ Thus, we need to consider a vast variety of physicochemical parameters that could describe the catalytic performance, and detailed atomic scale information is hard to be obtained from experimental studies, particularly under catalyst operating conditions. Additionally, there might be a higher complexity in real catalysis than what can be described by conventional theoretical modeling based on electronic structure calculations and statistical mechanics. In real catalysis, there might be not just one underlying process, but there is often a high intricacy of many underlying processes. Artificial intelligence (AI) may capture the catalytic progression better than previous theoretical/computational methods because it targets correlations and does not assume a single underlying physical model.

AI has been utilized in heterogeneous catalysis for discovering new catalytic materials and/or their design rules.^13−19^ AI can access correlations describing the measured target catalytic performance without explicitly modeling all the underlying phenomena. In particular, the Sure-Independence Screening and Sparsifying Operator (SISSO)^20,21^ has been adopted on data-centric approaches for heterogeneous catalysis.^22−25^ Analytical expressions describing the target catalytic performance are identified by SISSO. The expressions contain few key parameters, out of many offered parameters that characterize the materials and might be correlated with the underlying processes triggering, favoring, or hindering the performance. Those key descriptive parameters composing the SISSO expressions have been called materials genes,^22^ in analogy to genes in biology. Namely, the catalytic function of the material can be described by the combinations of the materials parameters analogously to how eye’s color and health characteristics are determined by the combination of genes. Crucially, SISSO can identify potentially nonlinear, intricate correlations between (high-quality) small data sets (e.g., hundreds of target values), and an immense amount of candidate analytic functions (e.g., millions) is considered in the analysis. This makes SISSO suitable for applications in heterogeneous catalysis, where obtaining a large amount of consistent experimental data (i.e., data generated according to consistent and reproducible procedures) is usually time- and resource-consuming. On the other hand, either experimental or theoretical parameters were used in the previous SISSO studies on heterogeneous catalysis.^22−25^ In the present study, we will combine these different types of data.

**Figure 1:**
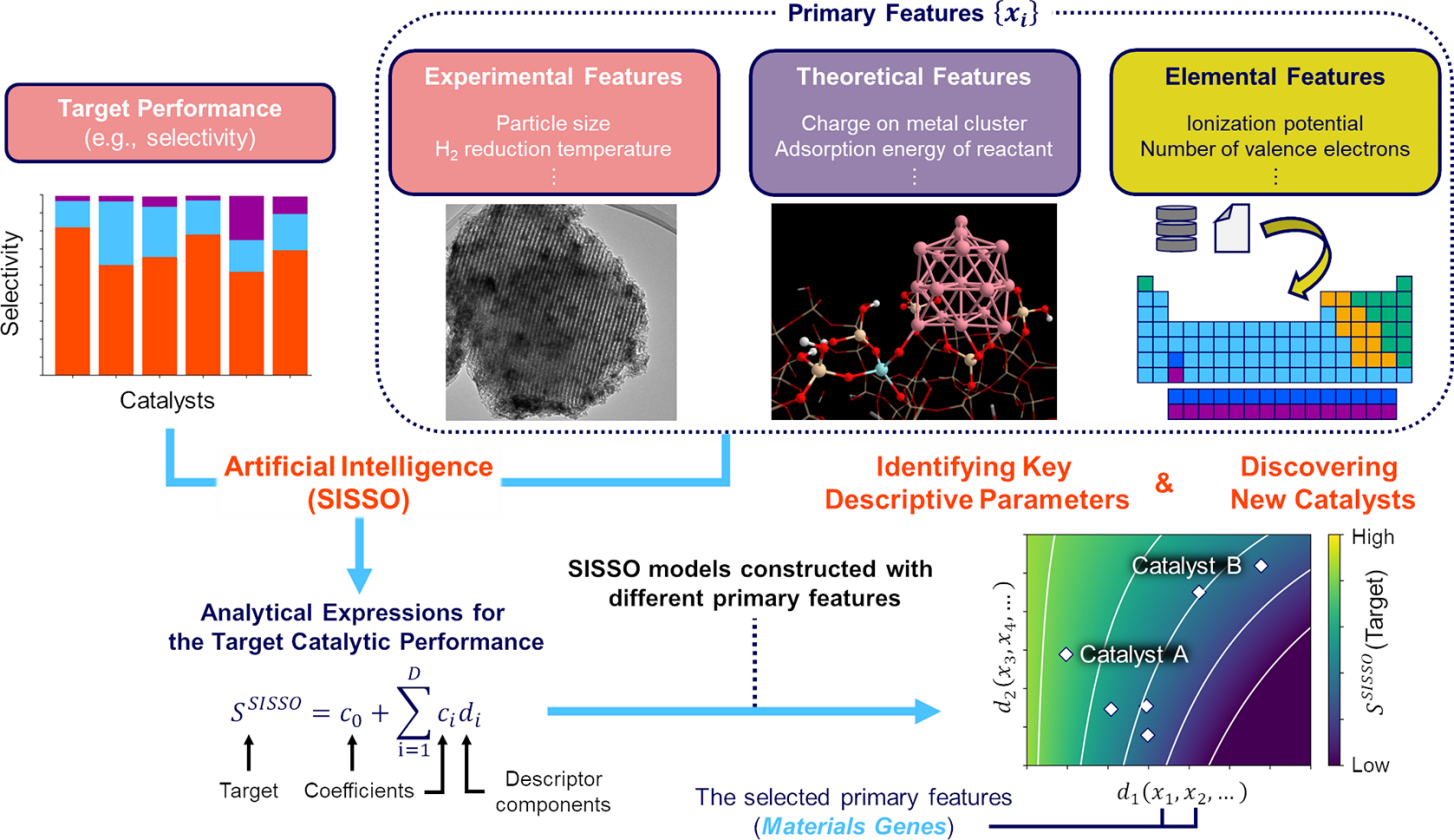
Schematic outline of the present study. By integrating information from different types of materials parameters (primary features) through the SISSO AI approach, we identify analytical expressions and the key descriptive parameters (materials genes) that are correlated to the measured (experimental) target catalytic performance. The SISSO models based on primary features with low acquisition costs also accelerate the discovery of new high-performance catalysts.

Here, we exploit experimental, theoretical, and elemental parameters (termed primary features) to efficiently model the catalytic performance and guide materials design (Figure 1). The experimental parameters consist of catalyst properties measured experimentally. The theoretical parameters consist of atomic-scale information obtained by density functional theory (DFT-RPBE) calculations with atomistic models, such as adsorption energies of intermediates and charge state of the catalysts. The elemental parameters are properties of the atoms composing the catalysts, such as ionization potential and atomic radius. These different types of parameters might capture different underlying processes, and they have different acquisition costs. In particular, the acquisition of elemental features does not require an experiment or a high-cost calculation with a large-scale atomistic model. Thus, the elemental parameters could provide AI models for predicting the performance of materials that were not yet synthesized or modeled with an atomistic simulation.

**Figure 2:**
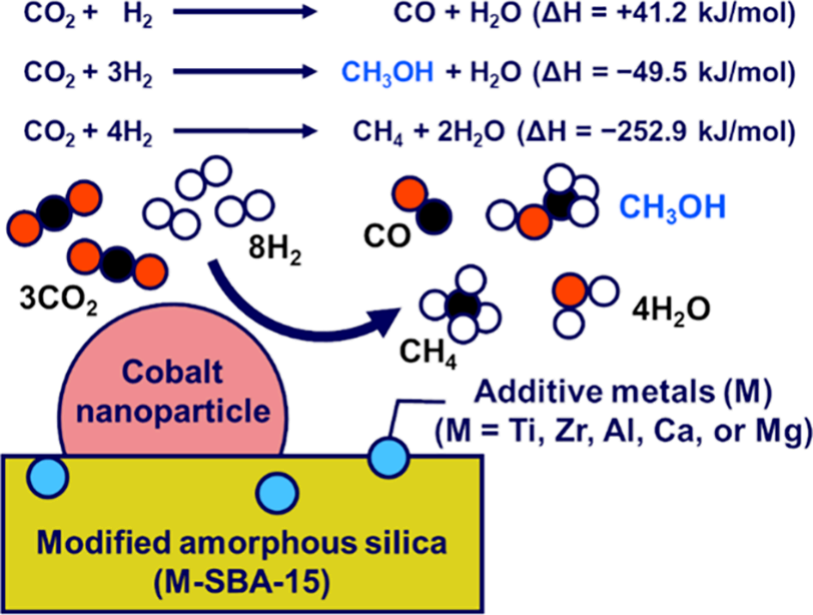
CO_2_ hydrogenation by the Co/M-SBA-15 catalysts. In addition to the gas-phase molecules shown in the figure, liquid-phase organic molecules (e.g., formate) are formed by the reaction.^26^ Formation enthalpies of the different reaction pathways (Δ*H* @ 298 K^35^) reflect the selectivity challenge in the CO_2_ hydrogenation to methanol. The catalytic performance of the Co/SBA-15 and Co/M-SBA-15 catalysts is shown in Figure S16.

We focus on CO_2_ hydrogenation catalyzed by cobalt nanoparticles supported on modified amorphous silica supports (Co/SBA-15 and Co/M-SBA-15 catalysts in Figure 2).^26^ Those catalytic systems mimic the environment of hydrothermal vents, which are mainly formed by silica-rich mixtures of serpentinized peridotite and mafic materials. These materials could contain several metals, such as Al, Ca, and Mg. On the other hand, the reaction carried out in the gas phase, whereas the environment of the hydrothermal vent presents an aqueous environment. The adopted experimental setup corresponds to conditions of reduced water activity, which prevent hydrolysis,^27^ and water is produced in our system via the reverse watergas shift reaction. Several organic molecules are formed by the CO_2_ hydrogenation on the Co/M-SBA-15 catalysts (Figure S16 in the Supporting Information). We particularly attempt to elucidate the role of the modified amorphous silica support (M-SBA-15), in which different additive metals (e.g., M = Ti or Al) are introduced. In CO_2_ hydrogenation catalysis, various roles of the support materials have been reported. For instance, the support can provide oxygen vacancies and isolated metal sites on its surfaces, and it can modify the electronic structure and shape of the supported metal particles, or a mixing (alloying) between metal oxide supports and the supported metals might take place.^4,28,29^ Indeed, in the studied systems, the selectivity depends on the type of incorporated additive metals (see Figure S16).^26^ However, detailed, realistic microscopic modeling of catalytic processes on supported catalysts is rather challenging. Thus, design criteria for efficient support materials toward the desired catalytic performance have not been established yet. In the present study, we elucidate the most relevant properties of the modified silica supports and underlying catalytic mechanisms that lead to the formation of CH_3_OH, CH_4_, and CO by identifying the key descriptive parameters that correlate with the selectivity toward thses molecules. Furthermore, new additive metals that can improve the CH_3_OH selectivity are explored by using the SISSO model built only on elemental features (Figure 1). Those results can contribute to understand the key environments and the underlying mechanisms for the metabolic intermediate generation. Additionally, the obtained materials genes and candidate additive metals can be utilized to design catalysts for CH_3_OH production via CO_2_ hydrogenation, which is a key catalytic reaction toward a sustainable society.^3,30−34^

## 2 Details of AI Approach

### 2.1 SISSO Approach

To identify the materials genes describing the CO_2_ hydrogenation catalysis, we adopt the SISSO AI analysis^20,21^ implemented in the SISSO++ code.^36^ Physicochemical parameters potentially correlated to the target catalytic performance (primary features) are used as input. In this work, experimental characterization or first-principles calculations are used to obtain these primary features characterizing the catalyst materials and possible underlying processes. Then, mathematical operators, such as addition, division, and multiplication, are applied to the primary features for generating an immense number (up to millions) of analytic functions (descriptor candidates). SISSO then selects a few descriptor candidates and weighting coefficients by using the sure-independence screening (SIS) and the *l*_0_ regularization. As a result, an analytical expression that shows the best correlation with the target performance (*S*) is obtained as a linear combination of the selected descriptors
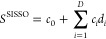
1

Here, *c*_0_ and *c*_*i*_ are the weighting coefficients, and *d*_*i*_ is the descriptor components. The primary features that appear in the expression of the descriptor components are the identified materials genes.

In this study, the multitask SISSO (MT-SISSO) transfer-learning approach^20^ is adopted. This approach generates a different SISSO model for each of the predetermined tasks, but the descriptor components of these different models are the same. Just the weighting coefficients are fitted to the training data points of each task (i.e., the weighting coefficients are functions of the tasks).

### 2.2 Primary Features and Target Performance

We consider experimental, theoretical, and elemental primary features to model the catalytic performance in CO_2_ hydrogenation. Experimental catalyst characterization primary features, simply denoted as the experimental primary features, are obtained from experimental characterization data of the Co/SBA-15 and Co/M-SBA-15 catalysts reported in our previous study.^26^ These features are materials properties, such as the surface area per catalyst weight (specific surface area) measured by N_2_ physisorption and the amount of desorbed CO_2_ measured by TPD (temperature-programmed desorption). The total number of experimental primary features is 15, and they are listed in Table 1. Note that we also adopt measured CO_2_ conversion () as an experimental primary feature to incorporate dependence of the CH_3_OH selectivity on the CO_2_ conversion. More details are discussed later in this section.

**Table 1:** Experimental Primary Features^a^

feature symbol	description	technique	unit
	CO_2_ conversion	GC	%
*T*_H_2__	temperature of the first H_2_ reduction signal	H_2_-TPR	°C
*W*_Co_	cobalt loading	SEM-EDX	wt %
	bulk M/Si ratio		-
	surface M/Si ratio		-
*S*_surf_	specific surface area	N_2_ physisorption	m^2^ g^–1^
*V*_pore_	total pore volume		cm^3^ g^–1^
*r*_pore_	mean pore diameter		Å
	amount of desorbed CO_2_ per mass	CO_2_-TPD	μmol CO_2_ g^–1^
	amount of desorbed CO_2_ per surface area		μmol CO_2_ m^–1^
	amount of consumed H_2_ per mass	H_2_-TPR	μmol H_2_ g^–1^
	amount of consumed H_2_ per surface area		μmol H_2_ m^–1^
ν_C_5_H_5_N_	difference of wavelength of pyridine FT-IR^b^	pyridine FT-IR	cm^–1^
*d*_Co_	mean cobalt particle diameter	TEM	Å

a Experimental primary features are based on the characterization schemes of ref (26). GC: gas chromatograph, TPR: temperature-programmed reduction, TPD: temperature-programmed desorption, SEM-EDX: scanning electron microscopy with energy dispersive X-ray analysis, FT-IR: Fourier transform infrared spectroscopy, TEM: transmission electron microscopy.

b Difference of absorption wavelength of the via vibrational mode between pyridine in the gas phase and pyridine adsorbed on the catalysts.

**Figure 3:**
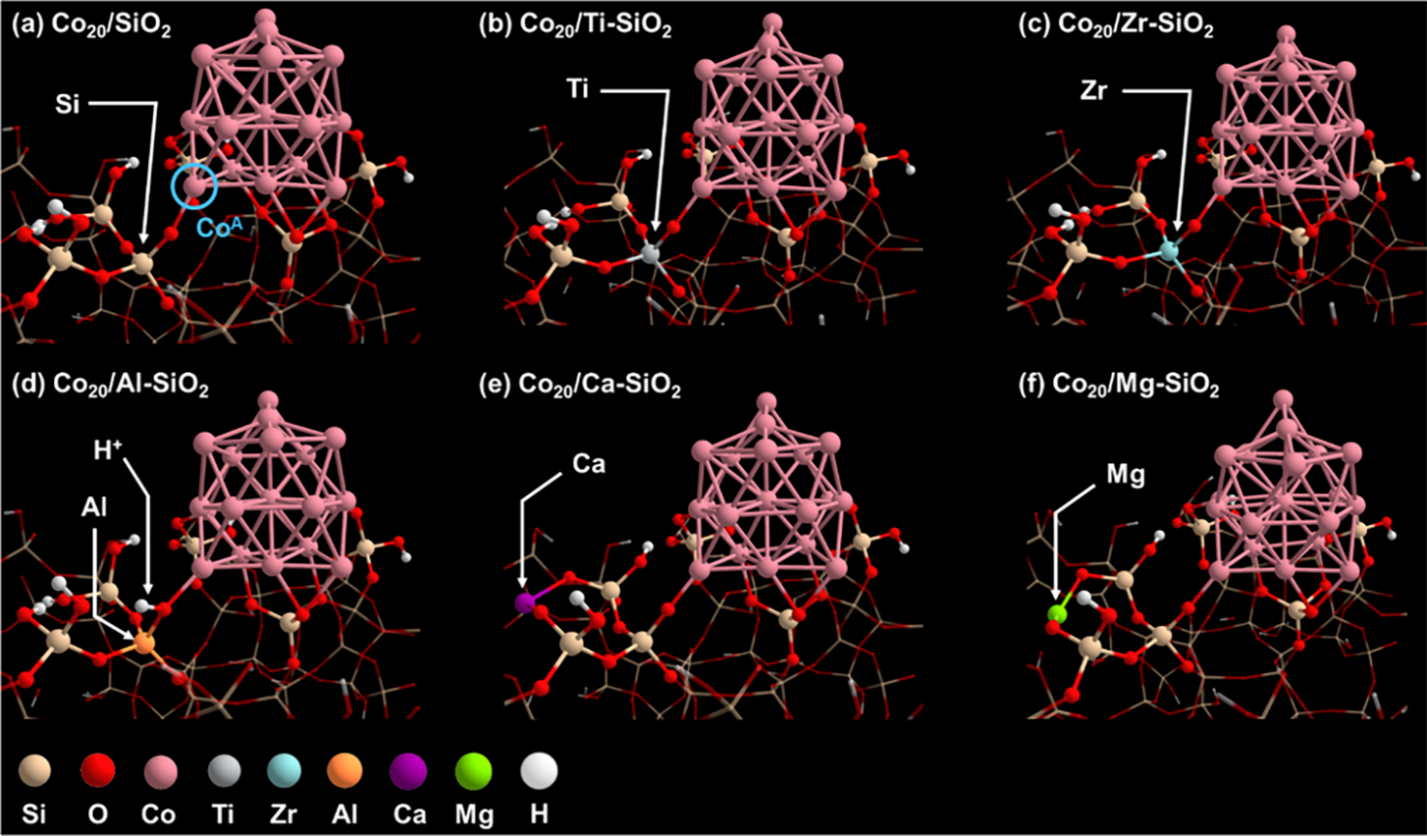
Co_20_/M-SiO_2_ models. The Co_20_ cluster and SiO_4_ that directly bond with Co_20_ and/or M are highlighted as the ball and stick model. The other parts are shown as the wireframe model. The Co_20_/SiO_2_, Co/Ti-SiO_2_, and Co_20_/Zr-SiO_2_ are reported previously in ref (26).

Theoretical catalyst characterization primary features*,* simply denoted as the theoretical primary features*,* are obtained by electronic-structure calculations using atomistic models. We use Co_20_/M-SiO_2_ models,^26^ where a Co_20_ cluster^37^ is supported on an amorphous silica surface slab,^38^ as theoretical models of the Co/SBA-15 and Co/M-SBA-15 catalysts (Figure 3). The additive metal in the support (i.e., Ti, Zr, Al, Ca, or Mg) is incorporated in the silica surface. The coordination numbers of these additive metals in the models are chosen based on their stable formal oxidation number (+4: Ti and Zr, + 3: Al, and +2: Ca and Mg). As reported in our previous study,^26^ a silicon atom in a SiO_4_ unit that directly interacts with the cobalt cluster is replaced by Ti or Zr (Co_20_/Ti-SiO_2_ and Co_20_/Zr-SiO_2_ models in Figure 3b,c, respectively). In the Co_20_/Al-SiO_2_ model (Figure 3d), the same silicon atom is replaced by Al, and a proton is incorporated in the AlO_4_ unit to keep charge of the system neutral with Al^3+^. The incorporated site of the proton shown in Figure 3d (i.e., – Al–O–Co−) is the most stable one compared with the other three sites in the AlO_4_ unit (i.e., −Al–O–Si−). Ca or Mg is incorporated in a bridge site between two SiO_3_–OH units by removing H atoms from OH groups in those units (Co_20_/Ca-SiO_2_ and Co_20_/Mg-SiO_2_ models in Figure 3e,f, respectively). We considered incorporating Ca or Mg into the bridge sites around the SiO_4_ unit where silicon is substituted by the additive metal in the other Co_20_/M-SiO_2_ models, and the most stable one is adopted. Although Ca and Mg are not directly interacting with cobalt in the atomistic model, the calculated values of the theoretical primary features, such as adsorption energies (Figures S3–S8 in the Supporting Information), are different from those obtained with the Co_20_/SiO_2_ model, where no additive metal is included. By incorporating Ca or Mg, the local structure around the cobalt cluster is modified, and this can in turn change the materials properties, including the stability of adsorbed species, for example, the most stable adsorption sites. Spin-polarized DFT calculations with the RPBE exchange–correlation functional^39^ were performed by using the FHI-aims code.^40^ More computational details are given in section S2.1 in the Supporting Information.

The theoretical primary features are materials properties as well as quantities reflecting the interaction of the catalysts with reaction intermediates of CO_2_ hydrogenation. For instance, the Hirshfeld charge of the additive metals in the silica support, the adsorption energies of the key species (e.g., CO_2_ and oxygen atom), the formation energies of the proposed intermediates for the CH_3_OH formation (e.g., HCOO and CH_3_OH), and the electronic and geometric structures of adsorbed CO_2_ (e.g., O–C–O angle) are included as the theoretical primary features. Ten theoretical primary features are used in total. They are listed in Table 2. We note that reconstructions and dynamical processes on the catalysts during the reactions are not considered in the Co_20_/M-SiO_2_ models. However, we hope that these processes, which are missing in our static DFT-RPBE calculations, are captured by the SISSO analysis. The goal of this work is not a fully theoretical explanation of the experimental findings but rather the identification of theoretical and experimental parameters that correlate with the measured performance. Indeed, our approach also includes experimental parameters that could correlate with reconstructions. For instance, the temperature of the first H_2_ reduction signal in TPR (temperature-programmed reduction) can capture the transitions between different oxide phases, which could affect the selectivity of the CO_2_ hydrogenation.^30,41−43^

**Table 2:** Theoretical Primary Features^a^

feature symbol	description	technique	unit
*E*_ads_^CO_2_^	adsorption energy of CO_2_ at the interfacial site	DFT-RPBE	eV
Δ_C–O_	sum of C–O bond elongation of adsorbed CO_2_^b^		Å
Δ_∠O–C–O_	degree of O–C–O angle bending of adsorbed CO_2_		degree
*q*_CO_2__	the Hirshfeld charge of adsorbed CO_2_		e
*q*_M_	the Hirshfeld charge of the additive metal		e
*E*_HCOO_	formation energy of HCOO at the interfacial site		eV
*E*_COOH_	formation energy of COOH at the interfacial site		eV
*E*_CO+O_	formation energy of CO + O at the interfacial site		eV
*E*_CH_3_O_	formation energy of CH_3_O at the interfacial site		eV
*E*_ads_^O^	adsorption energy of O atom at the interfacial site		eV

a Primary features obtained by Co_20_/M-SiO_2_ models. More details are given in section S2.1.

b Summation of elongations of two C–O bonds of the adsorbed CO_2_ compared to the bond lengths in the gas phase.

In addition to the experimental and theoretical primary features, we also employ elemental primary features that are atomic properties of the additive metals, such as ionization potential, number of valence electrons, or properties of atomic dimers reflecting the interaction of metal atoms with C, O, and H atoms. The eight elemental primary features are listed in Table 3.

**Table 3:** Elemental Primary Features

feature symbol	description	technique	unit
*M*_cov_	covalent atomic radius	exp. data^44^	Å
*MC*_rad_	radius of M^1+^	DFT-PBE0^45^	Å
*E*_M–C_	formation energy of a M–C dimer	DFT-PBE0^a^	eV
*E*_M–O_	formation energy of a M–O dimer		eV
*E*_M–H_	formation energy of a M–H dimer		eV
IP	ionization potential	exp. data^46^	eV
PEN	Pauling electron negativity	exp. data^47^	-
*N*_VE_	number of valence electrons	-	-

a More details are shown in section S2.2.

Our target quantity is the experimental CH_3_OH selectivity, *S*_CH_3_OH_^exp^, for six catalysts (Co/SBA-15 and Co/M-SBA-15 catalysts with M = Ti, Zr, Al, Ca, or Mg) under four different reactant gas space velocities (RGSV).^26^ These are velocities normalized with respect to the weight of the catalyst in the reactor. Within the MT-SISSO approach, each RGSV is treated as a different task, and six data points of CH_3_OH selectivity are included in each task. Thus, altogether we have 6 × 4 = 24 data points. Obviously, this is a very small number, and for most machine-learning approaches, this data situation would prevent a proper analysis. However, SISSO can identify potentially nonlinear, intricate correlations even in such challenging situation by offering an immensity of descriptor candidates. To avoid overfitting such small data set, the optimal complexity of the SISSO model with respect to its predictability is determined by cross-validation, as described in the next section.

**Figure 4:**
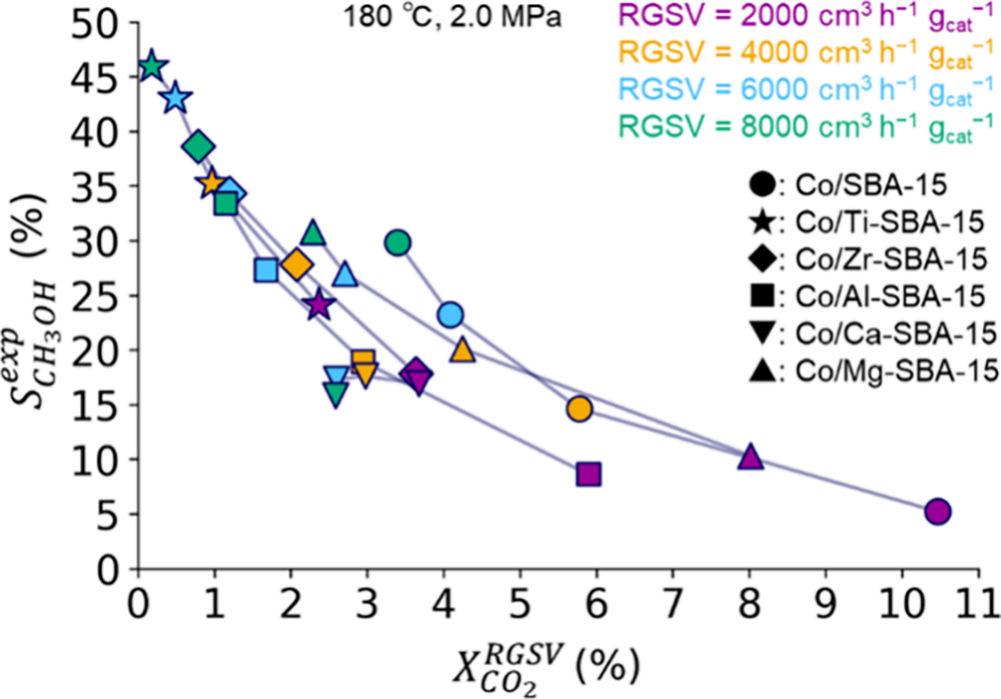
Experimental CH_3_OH selectivity () and CO_2_ conversion () of the Co/SBA-15 and Co/M-SBA-15 catalysts at different RGSV.^26^ The four colors label the different RGSV, and the six different shapes label the different catalysts. The lines connect RGSV values of the same catalysts. Reaction temperature and pressure are 180 °C and 2.0 MPa, respectively.

Figure 4 shows  and the experimental CO_2_ conversion () values for the different materials and RGSVs. Because the selectivity depends on the conversion level, the methanol selectivity should be analyzed by considering the different CO_2_ conversions achieved by the different catalysts. Thus, in the SISSO analysis, CO_2_ conversion is offered as an experimental primary feature to incorporate the selectivity dependency with respect to conversion. At RGSV = 4000 (cm^3^ h^–1^ g_cat_^–1^), where the selectivity and the CO_2_ conversion are in a reasonable balance,^26^ the most selective material is Co/Ti-SBA-15 with a selectivity value of 35.2% at the conversion of 1.0% (Figure 4). Co/Zr-SBA-15 shows the second highest CH_3_OH selectivity (27.8%), and the remaining materials have = 14.6–20.1%. The conversions are in the range of 1.0–5.8%, and Co/Ti-SBA-15 and Co/SBA-15 show the lowest and highest conversion, respectively.

### 2.3 Choice of SISSO Model Complexity

The complexity of SISSO models is controlled by the number of times the mathematical operators are applied (rung: *Q*) to generate the descriptor candidates and by the number of descriptor components in the SISSO model (dimension: *D*). Thus, *Q* and *D* are hyperparameters and need to be chosen carefully. By increasing the *Q* and/or *D*, the complexity of the SISSO models also increases, and the training errors can be improved. However, the prediction errors do not necessarily decrease with increasing the complexity of the models.^48^ Thus, the prediction errors commonly present a minimum at a certain *Q* and *D*.

In this study, the optimal *Q* and *D* with respect to predictability are determined by leave-one-material-out cross-validation (LOMO–CV).^22,23^ This allows us to capture the trade-off between under- and overfitting of the models. In the LOMO–CV approach, data points related to one specific catalyst (e.g., Co/Ti-SBA-15) are removed from the training data. Then, the CH_3_OH selectivity of the left-out material is calculated (predicted) by the SISSO model trained on the remaining data, and root-mean-squared error (RMSE) of the prediction on the left-out material is obtained (Figure S1). This procedure is performed for all catalysts, and the average of the obtained RMSEs (CV-RMSE) is evaluated. The complexity (i.e., combination of *Q* and *D*) that shows the minimum CV-RMSE is selected as the optimal one.

We obtained models for the CH_3_OH selectivity considering all combinations with *Q* = 1,2 and *D* = 1,2,3. Additionally, we compared five different primary feature sets, containing (i) only the theoretical features (denoted “Theo”), (ii) only the experimental features (denoted “Exp”), (iii) the theoretical and experimental features (denoted “Theo + Exp”), (iv) the theoretical, experimental, and elemental features (denoted “Theo + Exp + Elem”), and (v) only the elemental features (denoted “Elem”).

The number of candidate descriptors considered in our SISSO analysis is on the order of hundreds to millions (Table 4). For the models obtained with “Theo” and “Exp” primary feature sets, the optimal complexity identified by LOMO–CV is *Q* = 1 *D* = 1. Thus, in these cases, the optimal complexity is the lowest one considered. In contrast, the optimal *Q* or *D* is 2 for the remaining models (Table 4). These results indicate that only for the case of the last three primary feature sets a more complex model is also more predictive. Further details on the LOMO–CV are given in section S1 in the Supporting Information.

**Table 4:** Number of Considered Primary Features and Generated Candidate Descriptors in the SISSO Analysis and the Optimal Complexity of the Models Identified by the LOMO–CV

primary feature set	number of primary features	number of candidate descriptors for *Q* = 1	number of candidate descriptors for *Q* = 2	Optimal (*Q*, *D*)
Theo	10	235	50,485	(1, 1)
Exp	15	304	63,110	(1, 1)
Theo + Exp	25	923	576,677	(2, 1)
Theo + Exp + Elem	33	1724	2,162,827	(2, 1)
Elem	8	154	24,652	(1, 2)

## 3 Results and Discussion

### 3.1 Prediction Accuracy of SISSO Models with Different Primary Feature Sets

**Figure 5:**
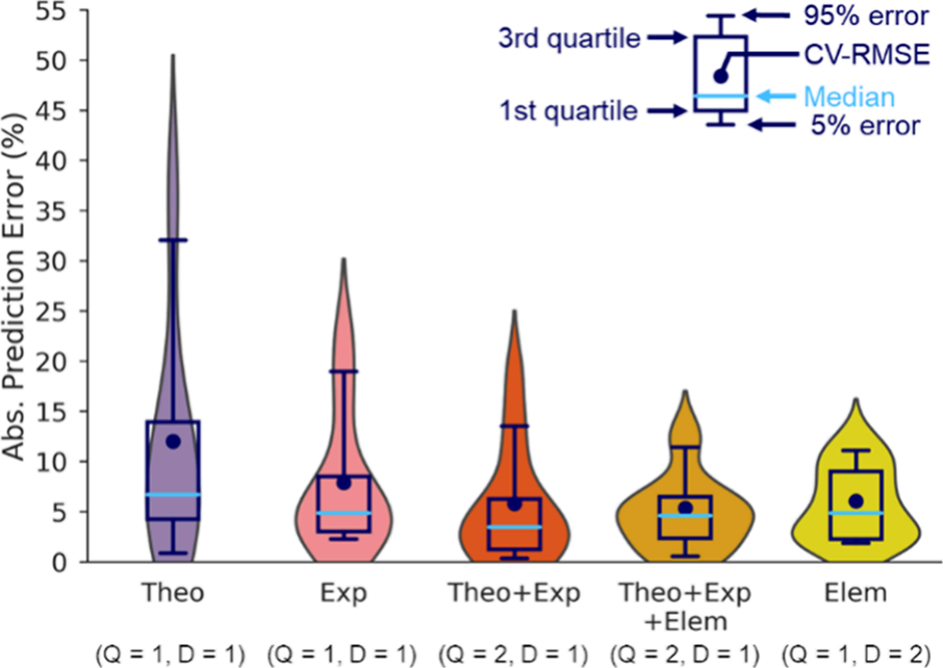
Box and violin plots of absolute prediction errors of LOMO–CV for SISSO models with different primary feature sets. The distribution of the prediction errors for the CH_3_OH selectivity shows how the combination of the different types of primary features improves the performance of the SISSO models. The upper and bottom whiskers of the box plots correspond to the 95th and 5th percentiles, respectively. The optimal complexity of each SISSO model (rung, dimension) is shown in parentheses.

Let us start with the analysis of prediction errors of SISSO models obtained for different primary feature sets at the optimal complexity. Figure 5 shows the box and violin plots of the absolute prediction errors, which correspond to the absolute CV errors on left-out materials (i.e., |Δ*M*_*i*_| in Figure S1). The width of the violin plots represents the density of data points with a certain error value. For the “Theo” and “Exp” models, which are built only with the theoretical or experimental primary features, respectively, the error distributions are broad (Figure 5). For example, their 95th percentiles of the prediction error distributions are 32.07 and 18.96%, respectively. Two catalysts have a particularly high prediction error for the “Theo” and “Exp” models. These are Co/Zr-SBA-15 and Co/SBA-15 (see Table S2). Thus, our offered primary features miss some of the relevant processes. When we offer experimental and theoretical primary features (“Theo + Exp” models), the error distribution becomes narrower, and the 95% error of this model decreases to 13.53%. In particular, Co/Zr-SBA-15 is now described well (Table S2). The quartile values (length of the box) and CV-RMSE are also improved (decreased). The results indicate that both experimental and theoretical primary features and their combinations are important to model and describe the experimental CH_3_OH selectivity of the CO_2_ hydrogenation. By further adding the elemental features along with experimental and theoretical features, a SISSO model with even lower prediction errors is obtained (“Theo + Exp + Elem” models in Figure 5). The 95% error of this model (11.43%) is now almost comparable with the standard deviation of the target calculated across the entire training set (10.48%). Note that the training error improves by increasing the number of the primary features because fitting gets better when more functions are offered. However, the prediction error may not improve because too many features increase the risk for causing overfitting.^48^ Thus, the improvement of the prediction error in the “Theo + Exp” and “Theo + Exp + Elem” with respect to “Theo” or “Exp” models is not only due to the increment of the number of primary features but also due to the fact that more processes governing the CH_3_OH selectivity are synergistically captured by the combination among features.

### 3.2 Investigation of the Materials Genes

Equation 2 is the SISSO model obtained with the theoretical, experimental, and elemental features (i.e., the “Theo + Exp + Elem” model) using the entire data set for training.
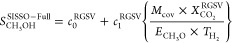
2

**Figure 6:**
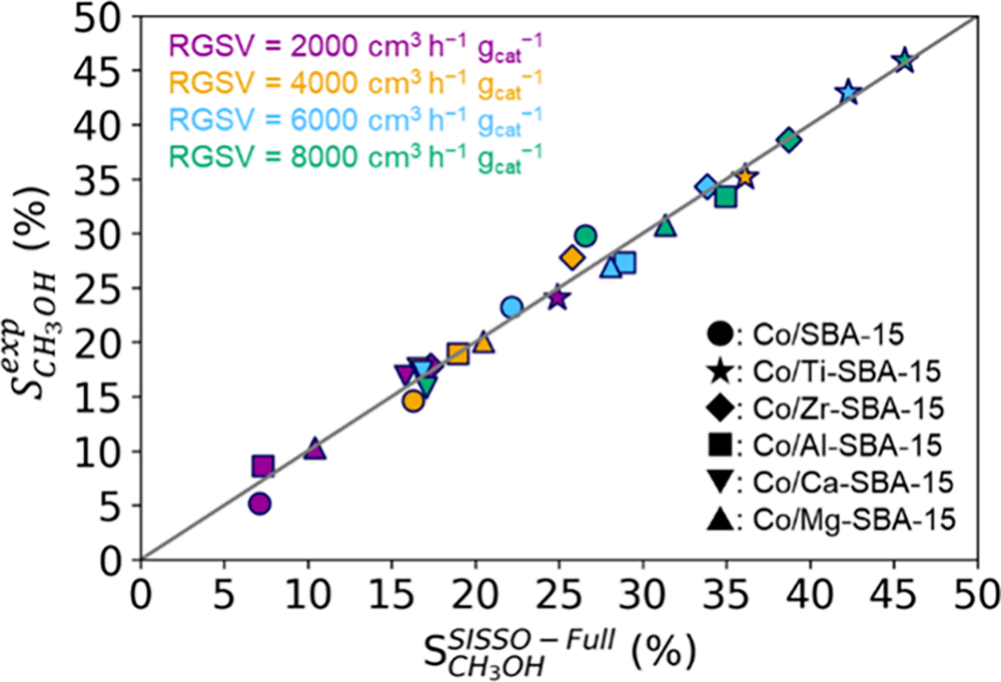
Comparison between the experimental selectivity results (*y*-axis) and the description provided by SISSO (*x*-axis). *g*_cat_ is the weight of the catalyst. The gray line shows the ideal relationship: = .

This model is based on a one-dimension descriptor. *M*_cov_ is the covalent atomic radius^44^ of the additive metals, and  and *T*_H_2__ are the experimental features, namely, the CO_2_ conversion and the temperature of the first H_2_ reduction signal. *E*_CH_3_O_ is the theoretical formation energy of CH_3_O intermediate at the interfacial site with respect to CO_2_ and H_2_ in the gas phase. The training RMSE of this model is 1.24%, and Figure 6 shows that the experimental CH_3_OH selectivity of each catalyst is well described.

The primary features that are selected by the SISSO model provide insights on the underlying processes. *T*_H_2__ can be assigned to reduction temperature for Co_3_O_4_ to CoO. Thus, *T*_H_2__ is related to the materials’ reducibility and its phase transitions. Indeed, those reconstructions can be crucial in cobalt-based systems for the CH_3_OH formation from CO_2_.^41^ reflects the dependence of the selectivity on the conversion. *E*_CH_3_O_ corresponds to the stability of CH_3_O, which is one of the key intermediates for the CH_3_OH formation as reported in previous studies about Cu catalysts supported on modified silica with highly dispersed hetero metal sites.^49−52^ Because CH_3_O is recognized as one of the final intermediates for the CH_3_OH formation, the stability of CH_3_O affects the selectivity. For instance, lower stability makes the reaction pathway for the CH_3_OH formation unfavorable, but too strong interaction between CH_3_O and cobalt causes poisoning of the certain (active) sites. *M*_cov_ corresponds to the size of the additive metals, and such difference of size might induce modification of the (local) structures of M-SBA-15. The shape of the cobalt particles and/or active sites might be affected by such structure difference of M-SBA-15.

We note that the conversion has been related to the amount of metallic cobalt in ref (26). We found correlations between  and the amount of consumed H_2_ per catalyst mass during TPR measurement (). The Pearson correlation coefficients between these features are close to the unity (0.95, see Figure S9a).  represents amount of reduced cobalt oxides by H_2_ and corresponds to amount of metallic cobalt (see Figure S9b). Namely, it is suggested that Co/SBA-15 has a large amount of the metallic cobalt compared to Co/Ti-SBA-15. These two features also correlate with amount of desorbed CO_2_ per catalyst mass during TPD measurement (), which reflects the amount of adsorbed CO_2_ on the catalyst.^26^ Thus, the metallic cobalt strongly binds CO_2_, but apparently, it is not a good species for CH_3_OH formation. On the other hand, cobalt silicate (Co–O–SiO_*x*_) at the interfacial region between cobalt and silica is hardly reducible species and could exist even after the reductive treatment in H_2_ (Figure S9b). Thus, Co/Ti-SBA-15 that shows a small  value has relatively large amounts of cobalt silicate. Therefore, those results suggest that cobalt silicate is one of the key species for the high CH_3_OH selectivity. A similar experimental observation was also reported by Wang et al.^42^ As observed above, the “Theo + Exp + Elem” model gives us the key materials properties that are the good starting points for further investigations toward the understanding of the catalytic mechanisms.

**Figure 7:**
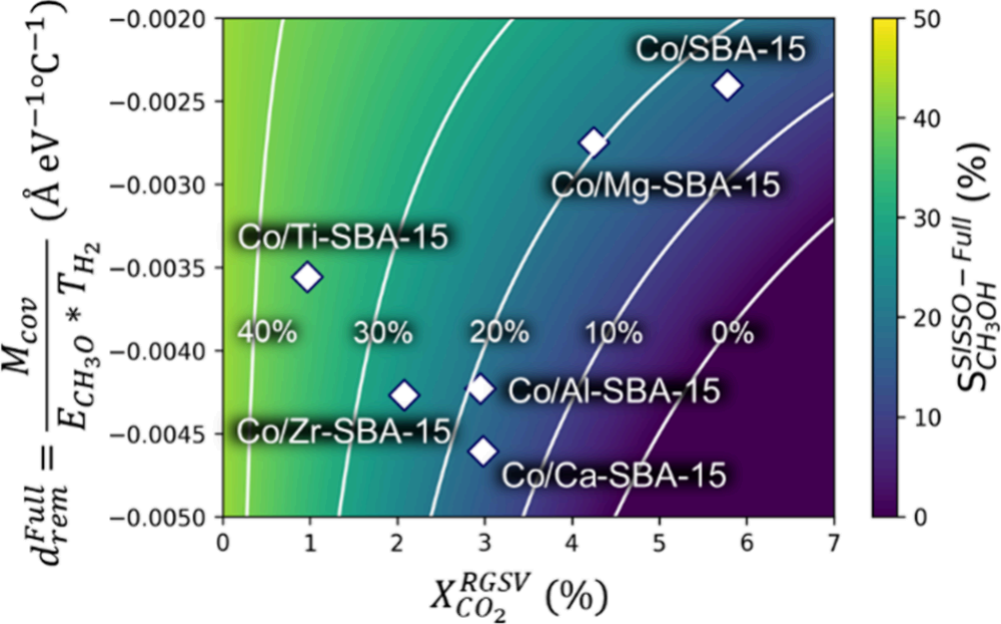
Catalyst map for the CH_3_OH selectivity focusing on  as the *x-*axis. The *y-*axis corresponds to the remaining part of the descriptor (). White squares are the data points of each catalyst. White lines correspond to contours (constant values) of CH_3_OH selectivity. In this figure, we focus on the values at RGSV = 4000 cm^3^ h^–1^ g_cat_^–1^. Note that the regions corresponding to  below 0% are colored with the same colors as for 0%.

To investigate how the identified materials genes correlate with the CH_3_OH selectivity, we show a “catalyst map” in Figure 7. In order to analyze the effect of materials properties and CO_2_ conversion separately, we chose  as the *x-*axis, and the *y-*axis is the remaining part of the descriptor of eq 2, . In this map, RGSV is set to 4000 cm^3^ h^–1^ g_cat_^–1^. The catalysts are distributed over the whole map. We also see that the CH_3_OH selectivity is inversely proportional to : The color in Figure 7 changes from yellow/green to blue/violet from left to right. For example, the catalyst with the highest CH_3_OH selectivity (Co/Ti-SBA-15) shows the lowest  and vice versa for Co/SBA-15 (Figures 4 and 7). On the other hand, an increase of  (the *y-*axis) improves the CH_3_OH selectivity. This trend is particularly pronounced in the high  region. Thus, not only the CO_2_ conversion but also  correlates with the CH_3_OH selectivity, and this result reflects the importance of combining different types of features and intricacy of describing the CH_3_OH selectivity.

The relevance of these parameters and the underlying processes that they capture would not be identified by a traditional theoretical analysis of the electronic structure via first-principles atomistic simulations or by an AI analysis with experimental data only (refs (22) and (23)) independently, but they could be unveiled by the strategy introduced in this paper. In particular, by using theoretical and experimental data with SISSO, we allow that microscopic calculated parameters and experimental mesoscopic parameters are combined in a single analytical expression to model the catalytic performance. This enables capturing the interplay or coupling of processes occurring at different length scales.

### 3.3 Materials Genes for the CH_4_ and CO Selectivity

SISSO models for the measured CH_4_ and CO selectivity of the Co/M-SBA-15 catalysts were also obtained. Equation 3 shows the expression of the “Theo + Exp + Elem” model for the CH_4_ selectivity. The optimal complexity identified for this target is *Q* = 2, *D* = 1 (see Figure S13b), with training- and CV-RMSE of 0.90 and 3.56%, respectively.
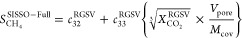
3

 and *M*_cov_ are selected as the materials genes for the CH_4_ selectivity. As discussed in the former section, those reflect the conversion dependence of the selectivity and the modification of the local structures of the catalysts, respectively. *V*_pore_ is the pore volume of the catalysts, and it can be related to transport of the reactant and/or products. As shown in Figure S13c, the CH_4_ selectivity increases as RGSV decreases. This suggests that the transport of the reactant species and the contact time between the reactant gas and the catalysts both affect the CH_4_ selectivity.

Equation 4 shows the expression of the “Theo + Exp + Elem” model for the CO selectivity. *Q* = 2, *D* = 2 is the optimal complexity for this model (see Figure S13e), with training- and CV-RMSE of 0.12 and 5.44%, respectively.
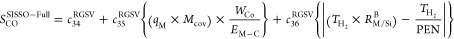
4

In eq 4, *q*_M_ and PEN are the Hirshfeld charge and the Pauling electronegativity of the additive metals, respectively. *E*_M–C_ is the formation energy of a M–C dimer and corresponds to the strength of the interaction between the additive metal and a carbon atom (see more details in section S2.2 in the Supporting Information). These three features can be related to the interaction between the additive metals and reaction intermediates such as CO. For instance, strength of the σ-donation and π-backdonation between CO and M can be correlated with those features.  is the bulk M/Si ratio, and it could be correlated with modification of the framework of SBA-15 and charge transfer between cobalt and SBA-15. *W*_Co_ is the cobalt loading. As discussed above, our SISSO approach can be applied for the different catalytic performances, and the identified materials genes can capture the relevant underlying processes for each considered target. Further details on the analysis of CH_4_ and CO selectivity are available in section S6 in the Supporting Information.

### 3.4 Exploring Other (New) Additive Metals by Using SISSO Models Trained with the Elemental Features

As discussed in the previous sections, the model obtained using experimental, theoretical, and elemental features describes the experimental CH_3_OH selectivity with reasonable accuracy. It is useful to investigate the details of the catalytic mechanisms. However, the model is not very helpful for predicting the CH_3_OH selectivity of new catalysts because we must synthesize such materials to obtain the experimental features needed for the evaluation of eq 2. Conversely, by using the SISSO model only with the elemental features (“Elem” model), exploration of new additive metals that improve the CH_3_OH selectivity can be accelerated because these features are readily available for a wide range of elements in the periodic table. Although the distribution of the quartile values of the prediction errors is broader than that of the “Theo + Exp” and “Theo + Exp + Elem” models (Figure 5), CV-RMSEs of the “Elem” model and those two models are comparable (CV-RMSE = 5.78, 5.35, and 6.05% for the “Theo + Exp”, “Theo + Exp + Elem”, and “Elem” models). The obtained “Elem” model is shown as eq 5.
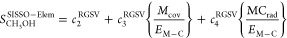
5

This model is based on a two-dimension descriptor. Similar to the “Theo + Exp + Elem” model, the covalent atomic radius^44^ of the additive metals (*M*_cov_) is also selected in the “Elem” model, suggesting that similar phenomena are being captured by this model. However, different materials genes are also identified by eq 5: MC_rad_ is the radius of the additive metal cations (M^1+^)^45^ and *E*_M–C_. MC_rad_ correlates with redox properties of the additive metals and might be related with the modification of SBA-15 structure explained in the previous section. *E*_M–C_ could correlate with stabilities of intermediates adsorbed (interacted) with the additive metal sites as also discussed in the former section.

**Figure 8:**
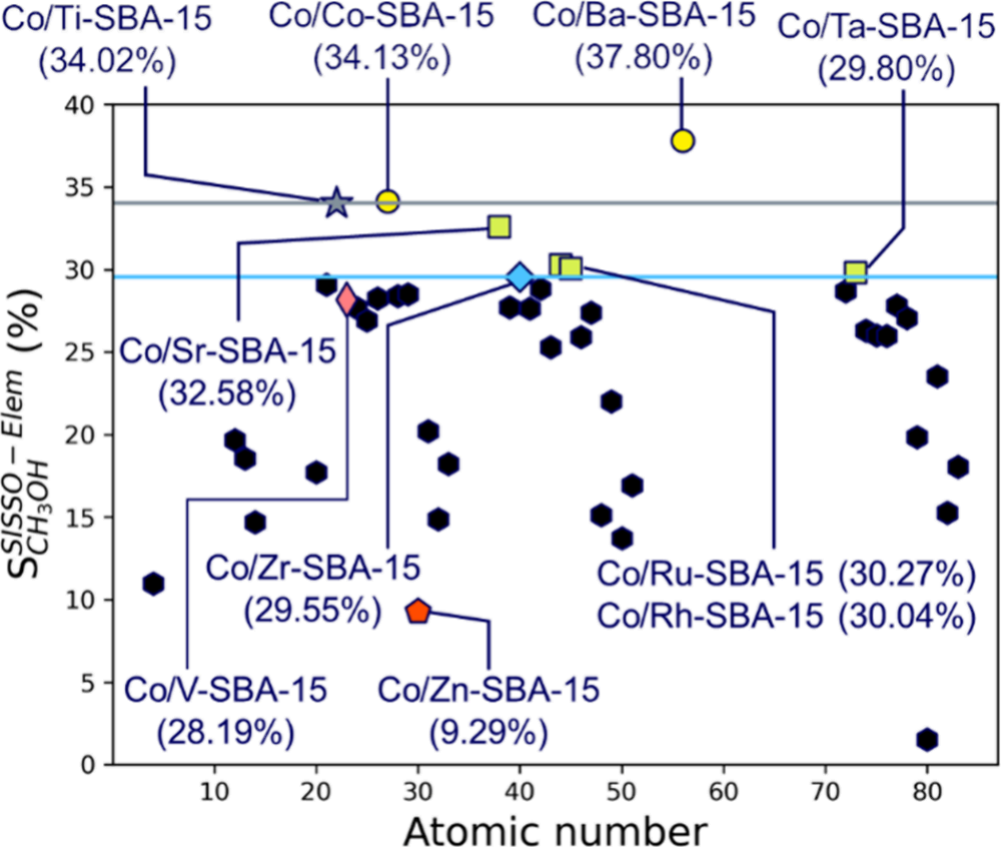
Predicted CH_3_OH selectivity by the “Elem” model. The *x-*axis represents atomic number of the additive metals. The gray and blue horizontal lines represent the CH_3_OH selectivity of Co/Ti-SBA-15 (34.02%) and Co/Zr-SBA-15 (29.55%) catalysts calculated by eq 5, respectively. In this figure, we focus on the CH_3_OH selectivity at RGSV = 4000 cm^3^ h^–1^ g_cat_^–1^.

**Figure 9:**
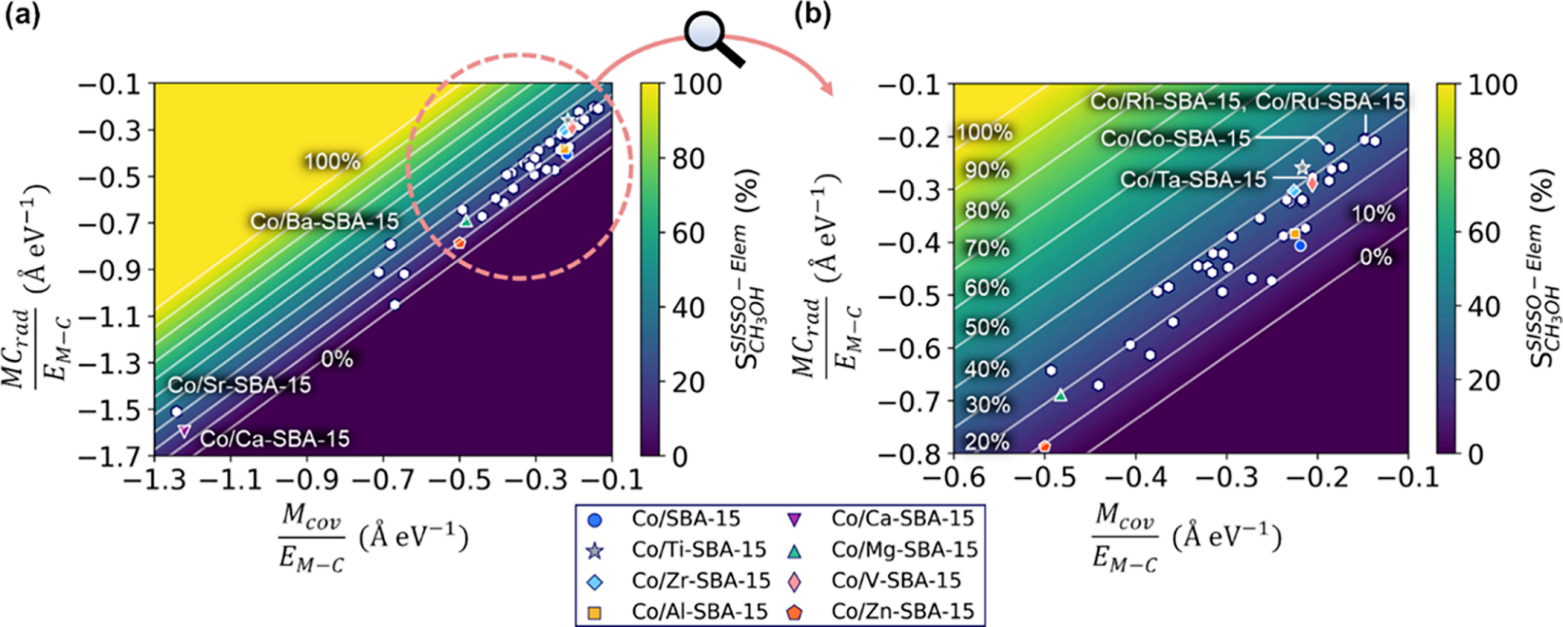
(a) Catalyst map for the CH_3_OH selectivity based on the “Elem” model. (b) The map focusing on the upper-right region (surrounded by a pink circle in panel (a)). The *x-* and *y-*axis of the maps are the descriptor components of the “Elem” model. Note that the regions corresponding to  above 100% and below 0% are colored with the same colors as for 100 and 0%.

By using eq 5, the CH_3_OH selectivity of the cobalt catalysts with new additive metals is calculated. In this study, we focus on metals in the groups 2–15 up to Bi excluding lanthanoids. As shown in Figure 8, Co/Co-SBA-15 and Co/Ba-SBA-15 (yellow circles) show higher CH_3_OH selectivity than that of Co/Ti-SBA-15 (gray star), which shows the highest CH_3_OH selectivity in the original experimental data set. Interestingly, cobalt itself is predicted as one of the best additive metals for improving the CH_3_OH selectivity. This result is in agreement with the discussion about the active sites for the CH_3_OH formation in the previous section, which is the cobalt species strongly interacted with silica (Co–O–SiO_*x*_). Additionally, we also find that Co/Sr-, Ru-, Rh-, and Ta-SBA-15 (green squares in Figure 8) show higher CH_3_OH selectivity than that of Co/Zr-SBA-15 (blue diamond), which shows the second highest CH_3_OH selectivity in the training data. As observed above, we predict the candidate additive metals that can improve the CH_3_OH selectivity by using the SISSO model only with the elemental features. We also create a catalyst map based on the “Elem” model. As shown in Figure 9, Co/Sr- and Ba-SBA-15 are located far from the other candidate additive metals that show the high selectivity. Thus, those catalysts possibly show unique mechanisms, and for instance, similarities between Co/Sr-SBA-15 and Co/Ca-SBA-15 are suggested by the map. On the other hand, Co/Ru-, Rh-, Co-, and Ta-SBA-15 are close to Co/Ti- and Zr-SBA-15, which are the high-performance catalysts in the original training data set.

From all the elements considered in our screening, we focus on Co/V- and Zn-SBA-15 because synthesis procedures of those support materials are reported.^53−55^ For these materials, we estimated the uncertainty of the prediction on the CH_3_OH selectivity by using SISSO models for LOMO–CV (hereafter CV models) that are trained without one materials in the original training data set (i.e., SISSO models w/o M_1–4_ in Figure S1). In Figure S10, the CH_3_OH selectivity of Co/V- and Zn-SBA-15 predicted by each of the CV models is shown. The distribution of the predicted values for Co/V-SBA-15 is narrower than that for Co/Zn-SBA-15 (standard deviation is 3.89 and 20.56%, respectively). More details are discussed in section S4 in the Supporting Information. We note that only six predicted values are considered in our uncertainty estimation. Ideally, we should estimate the uncertainty from a larger ensemble of models. However, we could only deal with the limited number of models via LOMO–CV due to the small size of the data set.

To investigate the prediction accuracy of the “Elem” model, we synthesized Co/V- and Zn-SBA-15 catalysts. We successfully incorporated V and Zn into SBA-15 by almost comparable M/Si ratio (∼0.1) with that of the other catalysts in the original training data set. Additionally, several experimental characterizations, such as N_2_ physisorption and SEM-EDX, also show reasonable results, and more experimental details about the synthesis are shown in section S7 in the Supporting Information. Note that we also tried to synthesize the Co/Ba-SBA-15 catalyst based on a previous work.^56^ However, we could only synthesize the Ba-SBA-15 support that has a small M/Si ratio (0.02) compared with the other catalysts. The CH_3_OH selectivity of Co/V- and Zn-SBA-15 at each RGSV is shown in Figure S12c together with that of the other catalysts, and the selectivity at RGSV = 4000 cm^3^ h^–1^ g_cat_^–1^ is 32.5 and 16.9% for Co/V- and Zn-SBA-15, respectively. The Co/V-SBA-15 shows the second highest selectivity among all eight tested materials. The predicted CH_3_OH selectivity by the “Elem” model is 28.19% for Co/V-SBA-15 and 9.29% for Co/Zn-SBA-15. Thus, the prediction errors of the “Elem” model for Co/V- and Zn-SBA-15 are 4.31 and 7.61%, respectively. The prediction error for Co/V-SBA-15 is within the range of the prediction accuracy of the “Elem” model estimated by LOMO–CV (Figure 5). However, the prediction error for the Co/Zn-SBA-15 is higher than the estimated accuracy. Those differences on the prediction errors between Co/V- and Zn-SBA-15 are indeed expected from the uncertainty estimated by the CV models. As discussed above and in section S4, the uncertainty for Co/Zn-SBA-15 is larger. Those results suggest that, for the present data set, the uncertainty of the SISSO models can be estimated by using the CV models, and the prediction errors for new materials can be estimated by the computed uncertainty before synthesizing the materials.

By adding the new experimental CH_3_OH selectivity and the elemental features of Co/V- and Zn-SBA-15 to the data set, we retrained and updated the “Elem” SISSO model. The updated “Elem” model (here after “ElemUp” model) is shown as eq 6.
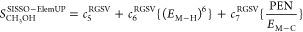
6

The optimal complexity of the “ElemUp” model is also *Q* = 1, *D* = 2 (see Figure S11). *E*_M–H_ is the interaction energy between a hydrogen atom and the additive metal. PEN is the Pauling electronegativity of the additive metals. The training- and CV-RMSEs are calculated to be 3.75 and 5.71%, respectively. As shown in Figure S12a, *E*_M–H_ is strongly correlated with *E*_M–C_, and PEN is negatively correlated with *M*_cov_ and MC_rad_. Thus, the materials genes newly selected in the “ElemUp” model and those in the “Elem” model possibly have similar roles on the descriptions for the CH_3_OH selectivity. To investigate the effect by the difference of the selected materials genes between the “Elem” and “ElemUp” models, a catalyst map of the “ElemUp” model is also investigated (Figure S12b). Compared with the map of the “Elem” model (Figure 9), Co/Zn-SBA-15 moves to be close to Co/Ca-SBA-15, and those catalysts are located away from the other catalysts. Interestingly, the CH_3_OH selectivity of those catalysts is almost independent of RGSV, and it is a unique trend in the given data set (Figure S12c). This can be an indication for a different reaction pathway compared to the other catalysts. The low number of training materials that display the RGSV dependency of the selectivity similar to that of the Co/Zn-SBA-15 material might contribute to the high prediction error on Co/Zn-SBA-15. On the other hand, in both maps, Co/V-SBA-15 is close to Co/Ti-SBA-15, and both catalysts show high CH_3_OH selectivity. This analysis illustrates how the description of the SISSO models can be updated by adding the new data points.

## 4 Conclusions

To identify the materials genes describing the CO_2_ hydrogenation catalysis, we employed the SISSO AI approach with primary features from experiments and computations. We focused on the CH_3_OH selectivity of the Co/M-SBA-15 catalysts, with different additive metals “M” in the support material. The prediction accuracy estimated by LOMO–CV is studied for different primary feature sets, and we obtain a model that well represents the experimental CH_3_OH selectivity by combining experimental + theoretical + elemental primary features. The selected primary features reflect the reducibility of cobalt species, the adsorption strength of reaction intermediates, and the chemical nature of the additive metal as important factors for the selectivity. To accelerate the exploration of new additive metals that improve the CH_3_OH selectivity, we also built a SISSO model only with the elemental features, which have low acquisition costs. Based on the predicted values by this model, we find new candidate additive metals that should have high CH_3_OH selectivity. We also synthesized cobalt catalysts with new additive metals, V and Zn. The SISSO model was then updated by adding the new experimental data of the Co/V- and Zn-SBA-15 catalysts. The differences of the selected materials genes in the original and the updated models were investigated by using the catalyst maps. Those results by our SISSO approach can be utilized not only to understand the mechanisms of the organic molecule generation at the early Earth but also to design CO_2_ conversion catalysts. We note that the generality of AI models is limited by the training data set. This also holds for SISSO though the interpretability of SISSO models and their physically meaningful primary features provide some hope for a good description. Importantly, by exploiting predicted metals and adding more data points, the generality of the SISSO models can be systematically improved.

## Supporting Information Available

Details of the SISSO approach and the DFT-RPBE calculations, correlation between the amount of consumed H_2_ and metallic cobalt, details of the “Elem” and “ElemUp” models, details of the SISSO models for the CH_4_ and CO selectivity, and the experimental details (PDF)
