# Supplementary material for: Materials Genes of CO2 Hydrogenation on Supported Cobalt Catalysts: An Artificial Intelligence Approach Integrating Theoretical and Experimental Data
Source: J Am Chem Soc. 2024 Feb 20;146(8):5433–44. doi: 10.1021/jacs.3c12984 (PMC10910553; doi:10.1021/jacs.3c12984)
Supplement: Supplementary file 1 — ja3c12984_si_001.pdf [file ja3c12984_si_001.pdf]

Supporting Information

Materials Genes of CO_2 Hydrogenation on Supported Cobalt Catalysts: an AI Approach Integrating Theoretical and Experimental Data

Ray Miyazaki,[1,†,*] Kendra S Belthle,[2] Harun Tüysüz,[2] Lucas Foppa,[1,*] and Matthias Scheffler[1]

[1]The NOMAD Laboratory at the Fritz-Haber-Institut of the Max-Planck-Gesellschaft and IRIS-Adlershof of the Humboldt-Universität zu Berlin, Faradayweg 4-6, 14195 Berlin, Germany.

[2]Max-Planck-Institut für Kohlenforschung, Kaiser-Wilhelm-Platz 1, 45470 Mülheim an der Ruhr, Germany.

[†]Present address: Institute for Catalysis, Hokkaido University, Sapporo 001-0021 Hokkaido, Japan

[*]Corresponding authors

TABLE OF CONTENTS

S1. Details of the SISSO approach

S2. Details of the DFT calculations

S3. Correlation between the amount of consumed H_2 and metallic cobalt.

S4. Details of the "Elem" model.

S5. Details of the "ElemUp" model.

S6. The "Theo + Exp + Elem" model for the CH_4 and CO selectivity

S7. Experimental details.

S1. Details of the SISSO approach

In this section, more details of the SISSO approach adopted in the present work and the SISSO models shown in Figure 5 in the main text are described. The optimal complexity (i.e., combination of rung (Q) and dimension (D)) of the SISSO models is determined by LOMO-CV (Figure S1). In this study, we consider all combinations within $Q = 1, 2$ and $D = 1, 2, 3$. The complexity that shows the minimum CV-RMSE is adopted as the optimal one. In the present study, we adopt the following mathematical operators: addition, (absolute) difference, multiplication, division, exponential, power (2, 3 or 6), square and cubic roots, and absolute value.

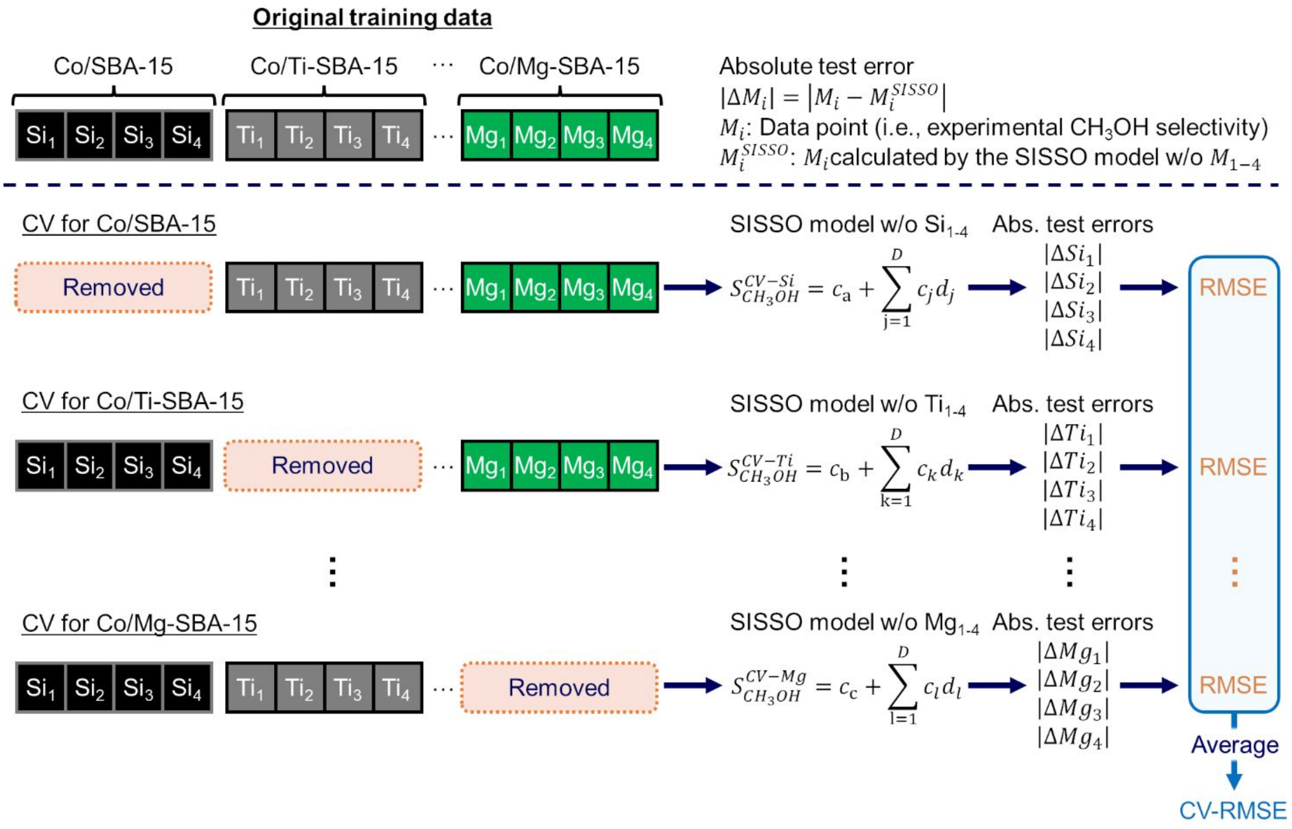

Figure S1. Leave-one-material-out cross-validation.

In Figure S2, CV-RMSE and RMSE for training (Training-RMSE) of the SISSO models shown in Figure 5 are summarized. The number of the descriptor candidates selected in the SIS process per dimension (N_{SIS}) is fixed to about the maximum number for constructing $Q = 1$, $D = 3$ model with the given primary feature set. For instance, about 210 descriptor candidates are generated from the theoretical features with $Q = 1$. Thus,

$N_{SIS} = 70$ is the maximum number for building $D = 3$ model with $Q = 1$. Because more descriptor candidates are generated with $Q = 2$ or 3, $N_{SIS} = 70$ is the upper limit to build the "Theo" models with the same N_{SIS} values in each complexity. The number of residuals used in the SIS process for the $D > 1$ models (N_{Res}), which is based on the multiple-residuals approach[1] implemented in the SISSO++ code,[2] is fixed to 5. The optimal complexity for the "Theo" and "Exp" models is $Q = 1$, $D = 1$ (eq S1 and S2, respectively).

$$S_{CH_3OH}^{SISSO-Theo} = c_8^{RGSV} + c_9^{RGSV}\left\{\left|E_{ads}^{O} - E_{ads}^{CO_2}\right|\right\} \quad ...(S1)$$

$$S_{CH_3OH}^{SISSO-Exp} = c_{10}^{RGSV} + c_{11}^{RGSV}\left\{\frac{X_{CO_2}^{RGSV}}{S_{surf}}\right\} \quad ... (S2)$$

For the "Theo + Exp" model, models with $Q = 1, D = 1$ and $Q = 2, D = 1$ show quite similar CV-RMSE (5.73% and 5.78%, respectively). Thus, the $Q = 2, D = 1$ model that shows lower Train-RMSE (1.32%) than that of the $Q = 1, D = 1$ model (2.69%) is adopted (eq S3).

$$S_{CH_3OH}^{SISSO-Theo+exp} = c_{12}^{RGSV} + c_{13}^{RGSV}\left\{\frac{X_{CO_2}^{RGSV}}{E_{HCOO}} * \frac{1}{\left(E_{ads}^{O}\right)^3}\right\} \quad ...(S3)$$

For the "Theo + Exp + Elem" model, $Q = 2$, $D = 1$ is adopted as the optimal complexity (eq 2 in the main text). For the "Elem" model, $Q = 1$, $D = 2$ is adopted as the optimal complexity (eq 5 in the main text).

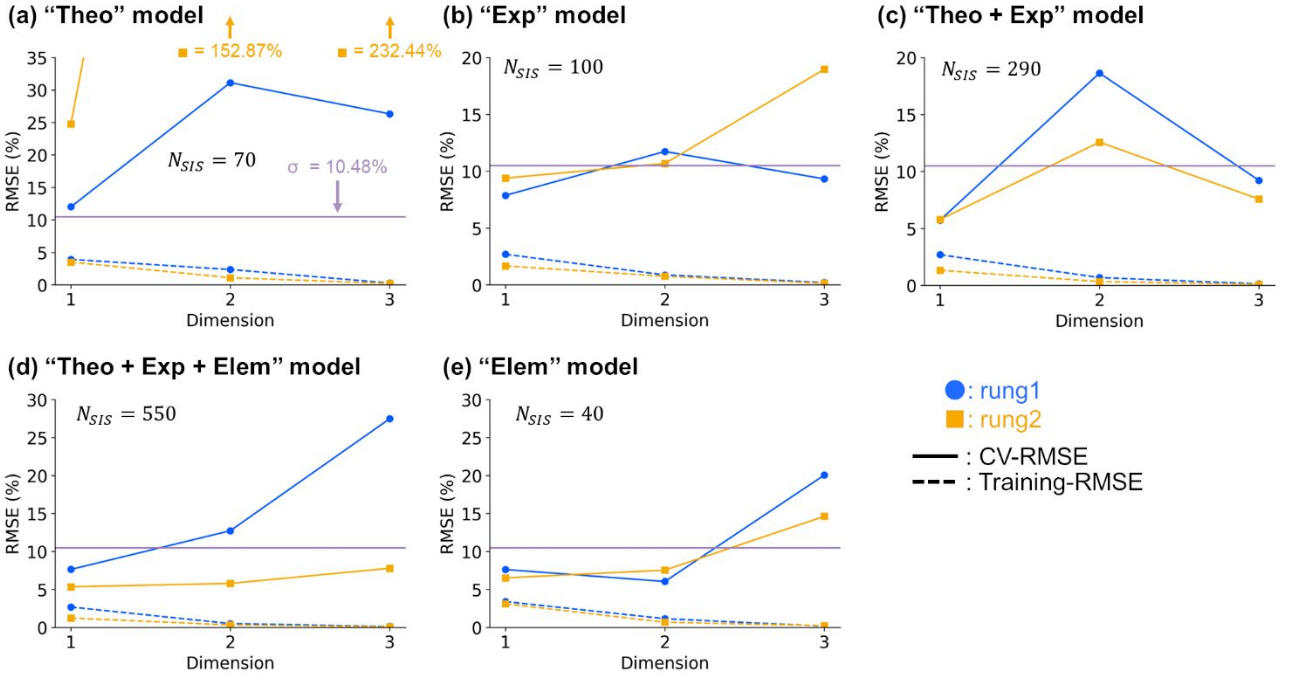

Figure S2. Training- and CV-RMSE curves of the SISSO models. A purple horizontal line corresponds to the standard deviation of the CH_3OH selectivity of the training data (σ = 10.48%).

Table S1. Coefficients of the definitive SISSO models trained by the whole data points with different primary feature sets.

Model	RGSV ($cm^3\ h^{-1}\ g_{cat}^{-1}$)	Coefficients		
		c_8^{RGSV}	c_9^{RGSV}	
Theo	2000	1.40E+02	4.87E+01	
	4000	2.18E+02	7.56E+01	
	6000	2.67E+02	9.21E+01	
	8000	2.91E+02	9.96E+01	
		c_{10}^{RGSV}	c_{11}^{RGSV}	
Exp	2000	2.62E+01	-1.17E+03	
	4000	3.54E+01	-2.14E+03	
	6000	4.14E+01	-3.00E+03	
	8000	4.43E+01	-3.33E+03	
		c_{12}^{RGSV}	c_{13}^{RGSV}	
Theo + Exp	2000	3.24E+01	-5.02E+01	
	4000	4.23E+01	-9.57E+01	
	6000	4.68E+01	-1.29E+02	
	8000	4.75E+01	-1.33E+02	
		c_0^{RGSV}	c_1^{RGSV}	
Theo + Exp + Elem	2000	3.38E+01	1.06E+03	
	4000	4.26E+01	1.90E+03	
	6000	4.66E+01	2.49E+03	
	8000	4.73E+01	2.53E+03	
		c_2^{RGSV}	c_3^{RGSV}	c_4^{RGSV}
Elem	2000	2.07E+01	-1.64E+02	1.28E+02
	4000	3.36E+01	-1.61E+02	1.33E+02
	6000	4.27E+01	-1.39E+02	1.22E+02
	8000	4.76E+01	-1.02E+02	9.75E+01

Table S2. Absolute prediction errors (%) of each SISSO model.[a] Those values correspond to $|\Delta M_i|$ in Figure S1 and are obtained by the CV-models.

Catalysts	RGSV (cm^3 h^{-1} g_{cat}^{-1})	Theo	Exp	Theo+Exp	Theo+Exp+Elem	Elem
Co/Si-SBA-15	2000	4.73	17.57	10.89	4.47	3.09
Co/Si-SBA-15	4000	0.51	18.96	13.53	6.54	6.08
Co/Si-SBA-15	6000	6.09	22.82	18.35	11.43	7.37
Co/Si-SBA-15	8000	11.11	23.10	19.13	13.17	7.34
Co/Ti-SBA-15	2000	12.12	3.94	0.10	2.49	9.71
Co/Ti-SBA-15	4000	13.92	6.30	1.36	5.11	10.60
Co/Ti-SBA-15	6000	14.82	6.97	4.51	2.02	11.31
Co/Ti-SBA-15	8000	13.12	4.23	2.38	0.55	9.55
Co/Zr-SBA-15	2000	22.77	3.06	3.62	0.60	1.62
Co/Zr-SBA-15	4000	32.07	1.41	2.26	2.53	2.70
Co/Zr-SBA-15	6000	38.66	2.49	3.35	0.57	4.24
Co/Zr-SBA-15	8000	38.18	2.49	3.10	0.17	3.70
Co/Al-SBA-15	2000	6.97	5.15	4.13	4.13	1.67
Co/Al-SBA-15	4000	5.24	5.27	5.36	5.36	1.95
Co/Al-SBA-15	6000	2.96	5.43	5.97	5.97	1.96
Co/Al-SBA-15	8000	0.18	4.44	4.66	4.66	1.86
Co/Ca-SBA-15	2000	11.06	2.93	0.82	0.82	11.12
Co/Ca-SBA-15	4000	2.09	7.69	2.94	2.94	2.30
Co/Ca-SBA-15	6000	6.46	10.98	7.11	7.11	5.52
Co/Ca-SBA-15	8000	14.00	14.77	12.63	12.63	12.38
Co/Mg-SBA-15	2000	0.90	4.57	0.39	6.55	3.55
Co/Mg-SBA-15	4000	2.86	3.81	0.29	6.46	2.21
Co/Mg-SBA-15	6000	4.92	2.27	1.04	4.77	6.35
Co/Mg-SBA-15	8000	5.64	2.11	0.35	4.61	8.87

[a]Values shown by orange correspond to the 95% error. Values shown by blue correspond to errors higher than the 95% error.

S2. Details of the DFT calculations

S2.1 The Co_{20}/M-SiO_2 models for the theoretical features

To obtain the theoretical features, the Co_{20}/M-SiO_2 models are adopted as theoretical models of the Co/SBA-15 and Co/M-SBA-15 catalysts. A Co_{20} cluster is supported on an amorphous silica surface slab model reported by Comas-Vives.[3] The most stable structure among 32 structures where the Co_{20} cluster is supported on the silica surface at the different interaction sites is adopted. More details about the model construction were reported in our previous study.[4] An additive metal (i.e., M = Ti, Zr, Al, Ca, or Mg) is also incorporated on the silica surface as discussed in the main text. We note that the Co_{20} cluster is smaller than the cobalt nanoparticles in the experiments (e.g., 8.8 nm for the Co/SBA-15 catalyst[4]). However, the results obtained with the Co_{20}/SiO_2 model were compared with a Co_{55}/SiO_2 model, which is reported as the minimum cluster size for representing small molecular adsorption on cobalt nanoparticles.[5] In both models, CO_2 prefers to be adsorbed on metallic cobalt rather than oxidized cobalt at the interfacial site, indicating that the trends captured by the small cluster agree with the results obtained in a large atomistic model (see more details in our previous study[4]).

The spin-polarized DFT calculations with the RPBE exchange-correlation functional[6] are performed by using the FHI-aims code (version: 210226).[7] Geometry optimizations are performed with the "light" basis set, and single-point energy calculations are performed with the "tight" basis set by using the optimized structures. A Γ-centered grid of 3 × 3 × 1 k-points is used for the calculations with the Co_{20}/M-SiO_2 models, and a single k-point is used for the isolated molecules in the gas phase (e.g., CO_2 and H_2). The atomic-scaled zeroth-order regular approximation (Atomic ZORA)[7] is adopted to incorporate relativistic effects. A ferro-magnetic ordering is used as the initial spin state of the Co_{20} cluster. The experimental lattice constants of bulk HCP cobalt are well represented by using those computational settings (deviation from the experimental values: $a = -0.01$ Å, $c = -0.03$ Å).[4] Additionally, the calculated vibrational frequency of the C−O stretching mode of the adsorbed CO on the Co(0001) surface with the $(\sqrt{3}\times\sqrt{3})R30°$ structure (1981 cm^{-1}) is in good agreement with the experimental value[8] (2015 cm^{-1}).

Adsorption energies (E_{ads}) of CO_2 and oxygen atom are calculated by the following equation.

$$E_{\mathrm{ads}} = E(\mathrm{Ads.} + \mathrm{Co_{20}/M\text{-}SiO_2}) - E(\mathrm{Co_{20}/M\text{-}SiO_2}) - E(\mathrm{Ads.}) \quad ...(S4)$$

Where $E(\mathrm{Ads.} + \mathrm{Co_{20}/M\text{-}SiO_2})$ represents potential energy of adsorption structure. $E(\mathrm{Co_{20}/SiO_2})$ and E (Ads.) represent potential energy of the Co_{20}/M-SiO_2 model and the adsorbate molecule in the gas phase, respectively. For adsorption energy of oxygen atom, $E(\mathrm{Ads.}) = 0.5 * E(O_2)$.

Formation energies (E_{int}) of the key intermediates for the CH_3OH formation are calculated by the following equation.

$$E_{\mathrm{int}} = E(\mathrm{Int.} + \mathrm{Co_{20}/M\text{-}SiO_2}) - E(\mathrm{Co_{20}/M\text{-}SiO_2}) - E(CO_2) - n * E(H_2) \quad ...(S5)$$

Where $E(\mathrm{Int.} + \mathrm{Co_{20}/M\text{-}SiO_2})$ represents potential energy of adsorption structure of the intermediate on the Co_{20}/M-SiO_2 model. $E(\mathrm{CO_2})$ and $E(H_2)$ represent potential energy of CO_2 and H_2 in the gas phase, respectively. For COOH and HCOO, $n = 1$. For CO+O, $n = 0$. Note that for HCOO and COOH, we calculate co-adsorption structure of the intermediate and a hydrogen atom to keep the model system neutral (see Figures S5 and S6). The formation energy of CH_3O is calculated by the following equation.

$$E_{CH_3O} = E(CH_3O + \mathrm{Co_{20}/M\text{-}SiO_2}) - E(\mathrm{Co_{20}/M\text{-}SiO_2}) - E(CO_2) - 2E(H_2) + E_{ads}^{O} \quad ...(S6)$$

Where $E(CH_3O + \mathrm{Co_{20}/M\text{-}SiO_2})$ and E_{ads}^{O} represent potential energy of adsorption structure of CH_3O and an oxygen atom (see eq S4) on the Co_{20}/M-SiO_2 model, respectively. Although co-adsorption structure of CH_3O and a hydrogen atom is considered in the model, an oxygen atom cleaved from CO_2 is not included (see Figure S8). Thus, adsorption energy of such oxygen atom is incorporated by adding E_{ads}^{O}.

In this study, we focus on a cobalt atom that directly connects to the MO_4 unit (Co^A in Figure 3a) and adsorption sites around Co^A. The adsorption site that shows the lowest E_{ads}, E_{int}, or E_{CH_3O} is adopted. The adopted adsorption structures of CO_2, O, and the key intermediates are shown in Figures S3-S8. We also consider the additive metals as the adsorption site. However, in some cases (e.g., HCOO on Ti site), we could not find such structure (i.e., the adsorbates are desorbed from the M site). Thus, we focus on the adsorption structures at around the Co^A site. Additionally, we also consider 3-coordinated Si, Ti, Zr, or Al structures. However, such structures are quite unstable (> 2.5 eV) compared with the 4-coordinated structure. Thus, we adopt the 4-coordinated structures.

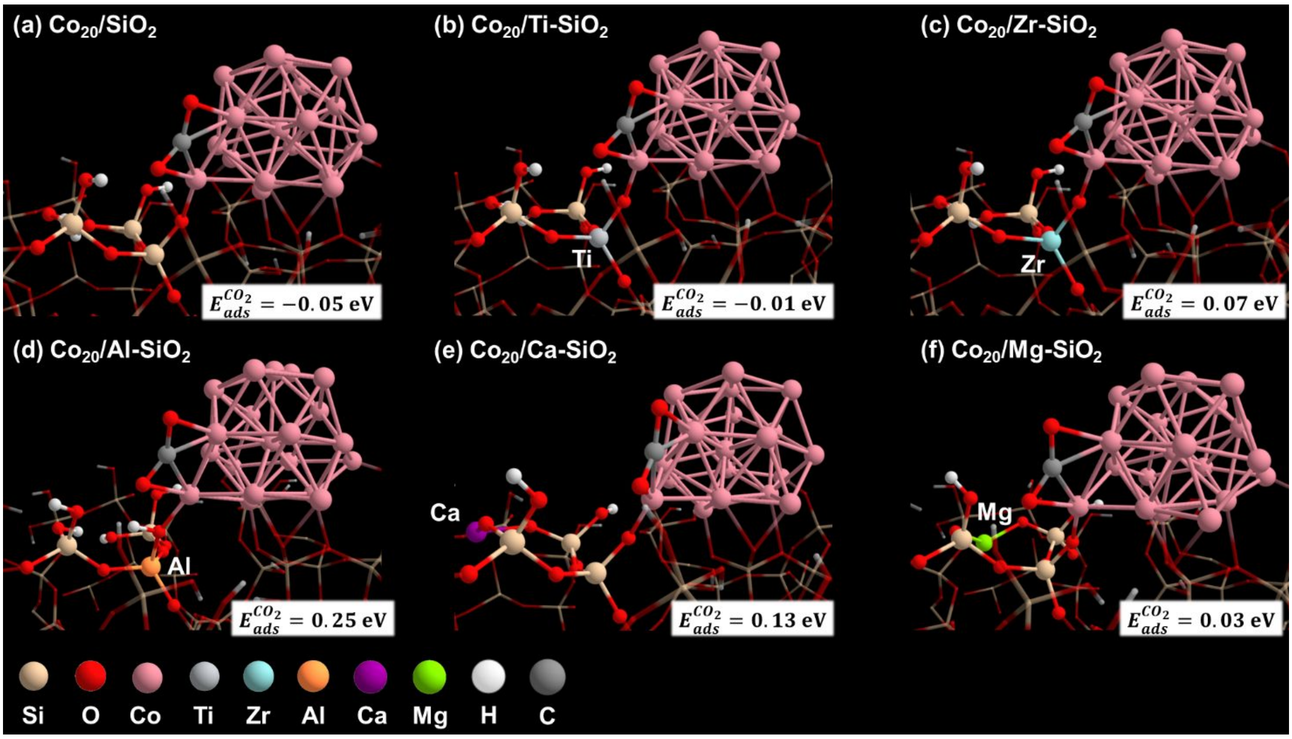

Figure S3. Adsorption structures of CO_2 on the Co_{20}/M-SiO_2 models.

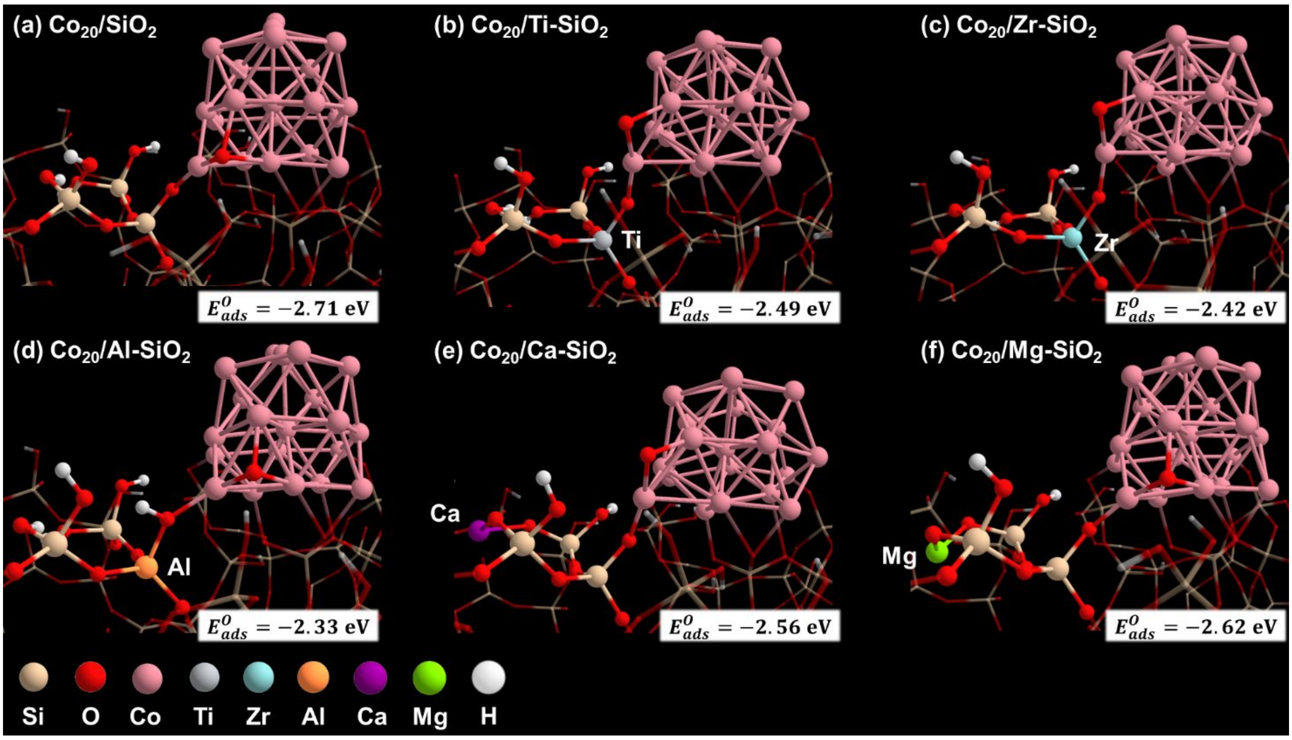

Figure S4. Adsorption structures of O on the Co_{20}/M-SiO_2 models.

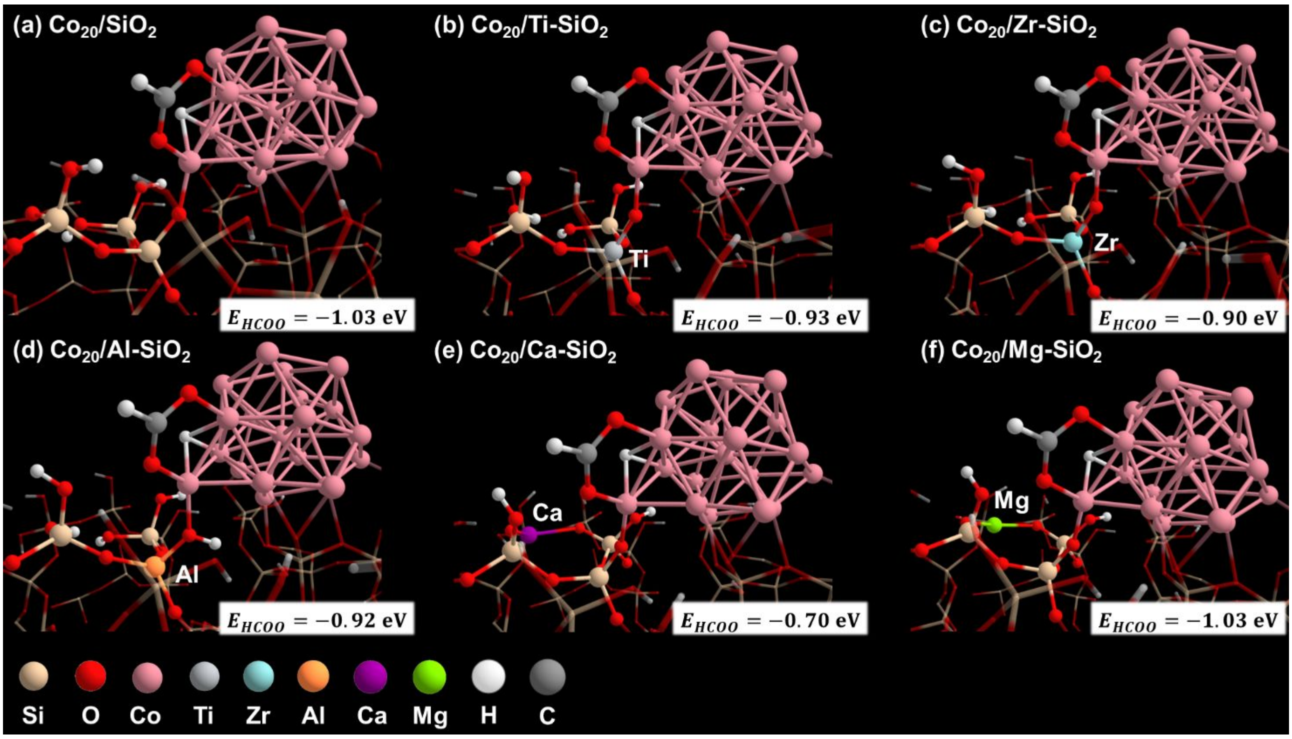

Figure S5. Adsorption structures of HCOO + H on the $Co_{20}/M\text{-}SiO_2$ models.

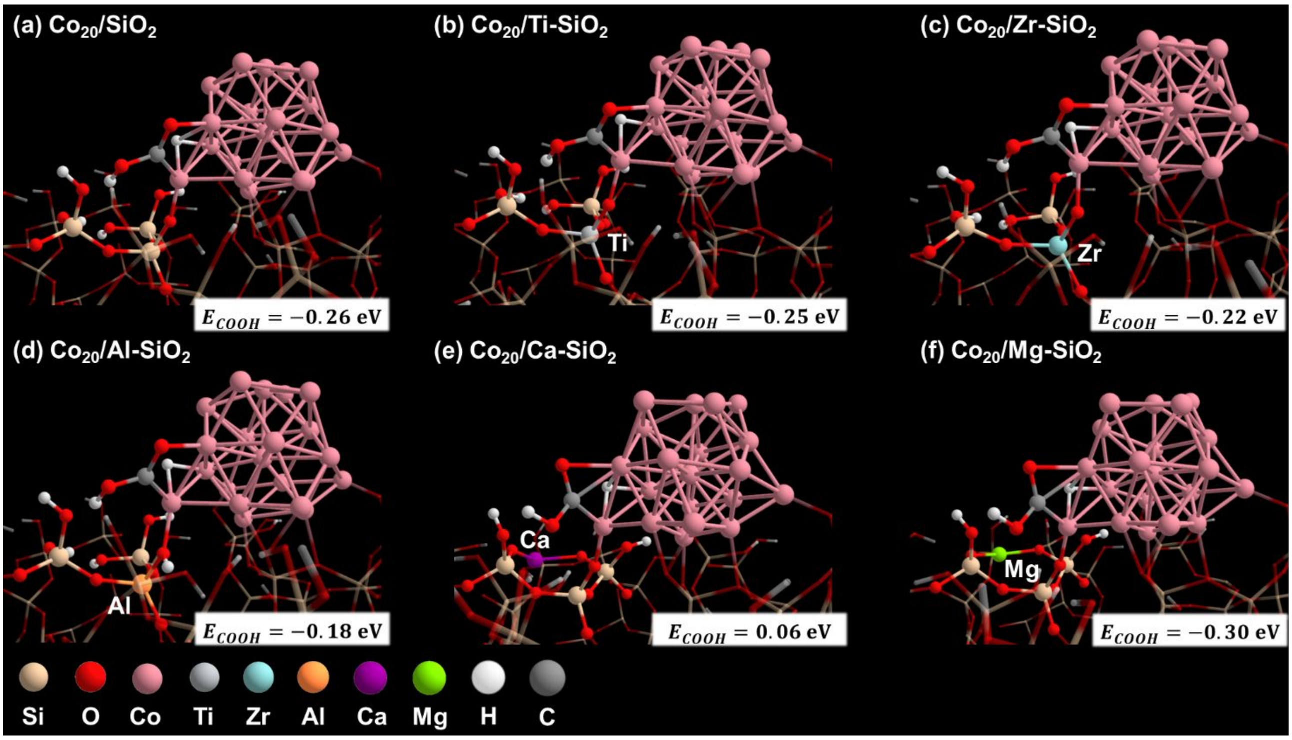

Figure S6. Adsorption structures of COOH + H on the $Co_{20}/M\text{-}SiO_2$ models.

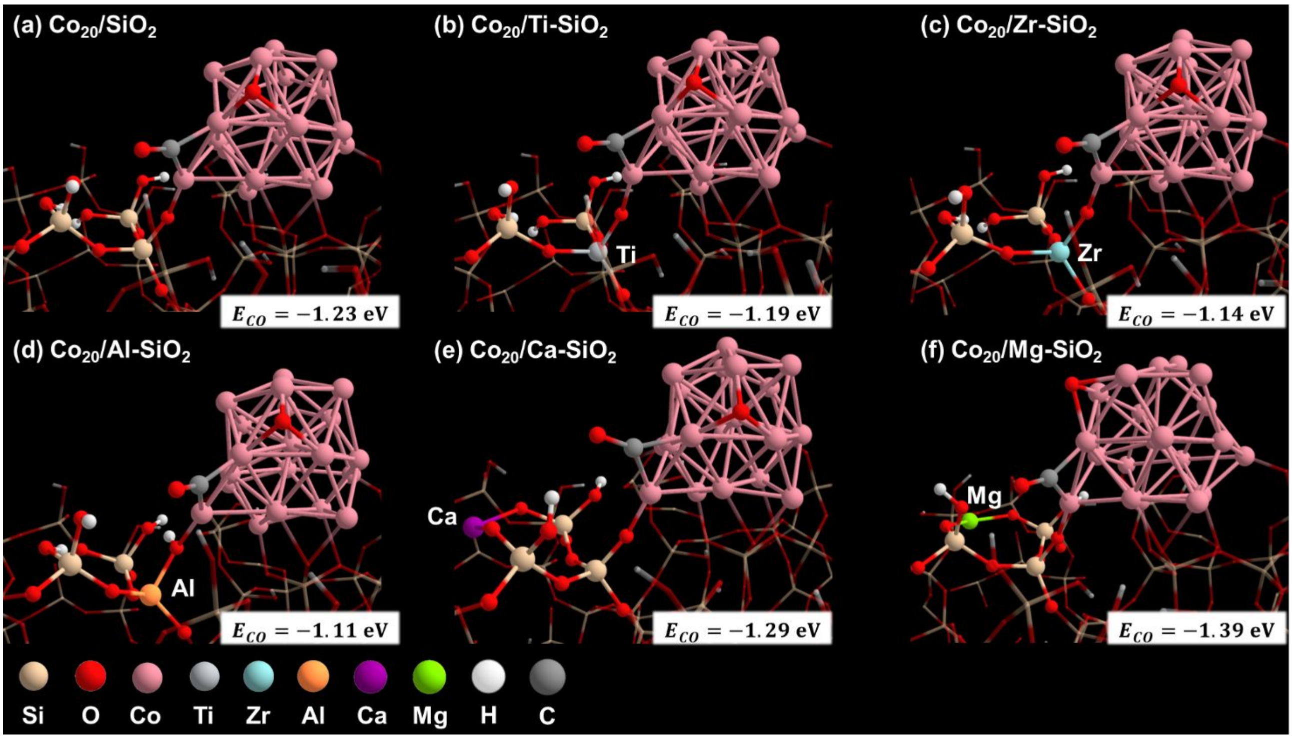

Figure S7. Adsorption structures of CO + O on the Co_{20}/M-SiO_2 models.

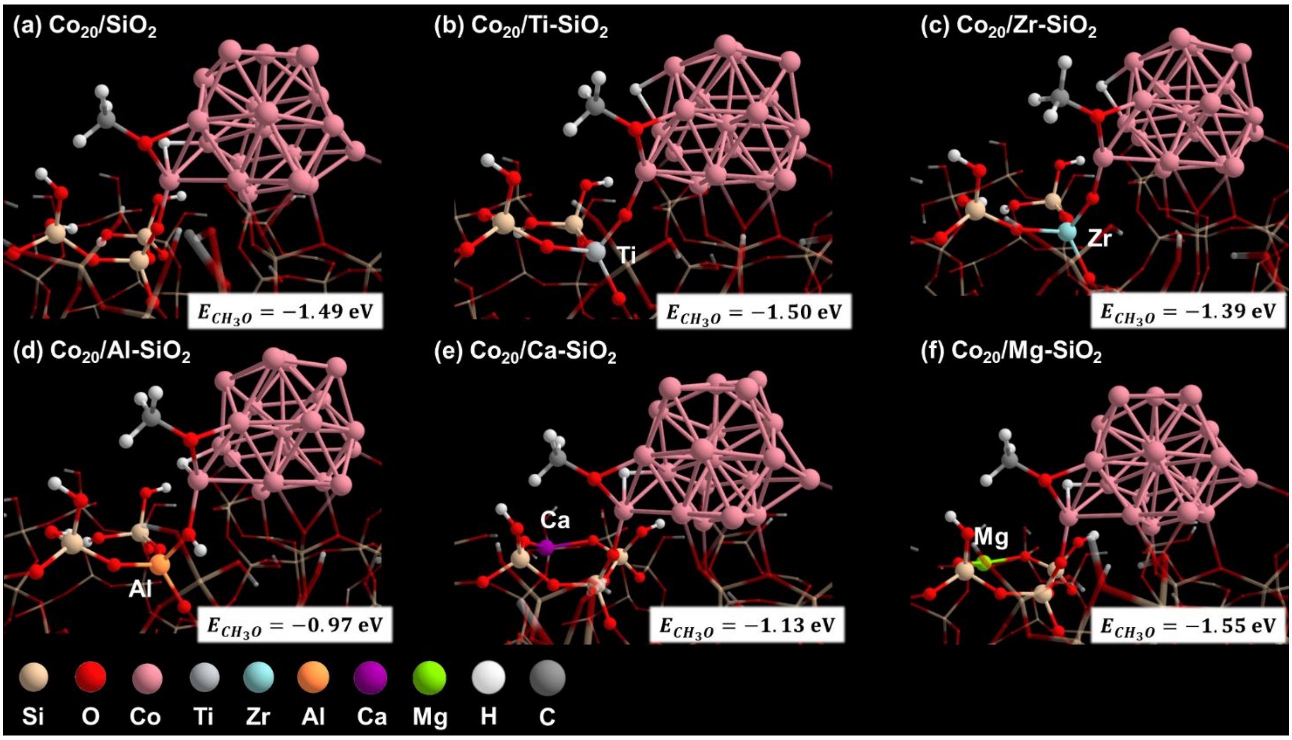

Figure S8. Adsorption structures of CH_3O + H on the Co_{20}/M-SiO_2 models.

S2.2 The M−X dimer models for the elemental features

To incorporate interaction energy between the additive metal and C, H, or O atom, formation energies of M−X dimers (X = C, H, or O) are calculated by the following equation.

$$E_{\mathrm{M-X}} = E(M\text{-}X) - E(M) - E(X) \quad ...(S7)$$

Where $E(M\text{-}X)$, $E(M)$, and $E(X)$ are potential energies of the M−X dimer, the additive metal atom, and X (= C, H, or O) in the gas phase, respectively. To obtain $E_{\mathrm{M-X}}$, the PBE0 exchange-correlation functional[9] and the "tight" basis set implemented in the FHI-aims code[7] are adopted for both geometry optimizations and the single-point energy calculations. A single k-point and a 50 Å × 50 Å × 50 Å unit cell are used. Low- and high-spin state are considered as the initial spin structure (even electron system: singlet and triplet, odd electron system: doublet and quartet), and more stable one is adopted. The other computational settings are same as written in section S2.1.

S3. Correlation between the amount of consumed H_2 and metallic cobalt.

Cobalt oxide can be reduced to metallic cobalt within the temperature range adopted in the H_2-TPR or the reduction. On the other hand, cobalt silicate is hardly reducible species, and thus, it is not reduced by those H_2 reduction treatments (see more details in our previous study[4] and its references). Therefore, the amount of consumed hydrogen during the H_2-TPR is negatively correlated with the amount of cobalt silicate (Figure S9b). In other words, the amount of consumed hydrogen is correlated with the amount of the metallic cobalt. Note that we assume that only three species (i.e., cobalt oxide, cobalt silicate, and metallic cobalt) exist in the cobalt nanoparticle before the H_2-TPR, and the cobalt oxide is completely reduced to the metallic cobalt by the TPR.

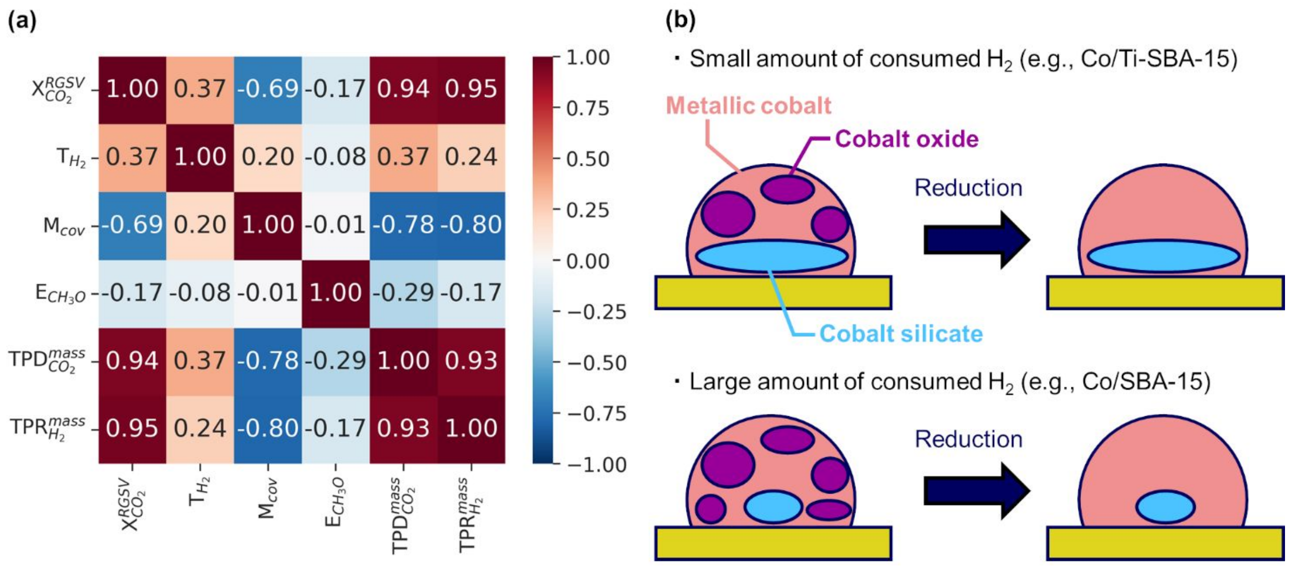

Figure S9. (a) The Pearson correlation matrix between the primary features. In this figure, we focus on the values at RGSV = 4000 cm^3 h^{-1} g_{cat}^{-1}. (b) Schematic picture for the correlation between the consumed H_2 and metallic cobalt.

S4. Details of the "Elem" model.

Uncertainty for the CH_3OH selectivity predicted by the "Elem" model is estimated by using the CV models (Figure S10). For Co/V-SBA-15, the predicted value by the definitive model that is trained on the entire dataset at the optimal complexity identified by LOMO-CV (eq 5) is close to the median of the predicted values by the CV models (29.41 %). On the other hand, the predicted value by the definitive model for Co/Zn-SBA-15 is lower than the median of the values by the CV models (18.04%). This result suggests underestimation by the definitive model for the prediction on Co/Zn-SBA-15. Additionally, for Co/Zn-SBA-15, the selectivity predicted by the SISSO model trained without the data points of Co/Ti-SBA-15 is highly deviated from that of the other CV models. Because Co/Ti-SBA-15 shows the highest CH_3OH selectivity in the original training data set, those data points are crucial to describe the trend of the target.

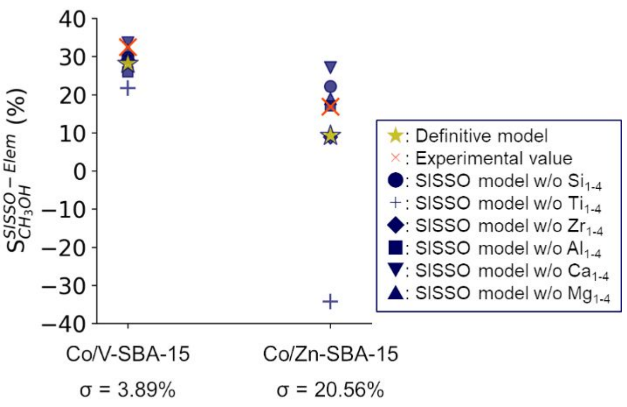

Figure S10. Uncertainty estimation on the CH_3OH selectivity prediction by the "Elem" model at RGSV = 4000 cm^3 h^{-1} g$_{cat}^{-1}$. Distribution of the predicted values by the CV models (blue scatters) is small for Co/V-SBA-15, but that for Co/Zn-SBA-15 is large. Standard deviation of the blue scatters is shown by σ. Such difference on the estimated uncertainty would be related to the prediction error of the definitive model trained by the all-data points (yellow star) for the corresponding experimental value (red cross).

The CV models of the "Elem" model are shown as eq S8-S13 (definition of $S_{CH_3OH}^{CV-M}$ is shown in Figure S1).

$$S_{CH_3OH}^{CV-Si} = c_{14}^{RGSV} + c_{15}^{RGSV}\left\{(M_{cov})^2\right\} + c_{16}^{RGSV}\left\{\frac{MC_{rad}}{PEN}\right\} \quad ...(S8)$$

$$S_{CH_3OH}^{CV-Ti} = c_{17}^{RGSV} + c_{18}^{RGSV}\left\{\frac{N_{VE}}{E_{M-C}}\right\} + c_{19}^{RGSV}\left\{(M_{cov})^3\right\} \quad ...(S9)$$

$$S_{CH_3OH}^{CV-Zr} = c_{20}^{RGSV} + c_{21}^{RGSV}\left\{\frac{M_{cov}}{E_{M-C}}\right\} + c_{22}^{RGSV}\left\{\frac{MC_{rad}}{E_{M-C}}\right\} \quad ...(S10)$$

$$S_{CH_3OH}^{CV-Al} = c_{23}^{RGSV} + c_{24}^{RGSV}\left\{\frac{M_{cov}}{E_{M-C}}\right\} + c_{25}^{RGSV}\left\{\frac{M_{cov}}{MC_{rad}}\right\} \quad ...(S11)$$

$$S_{CH_3OH}^{CV-Ca} = c_{26}^{RGSV} + c_{27}^{RGSV}\left\{E_{M-H} * PEN\right\} + c_{28}^{RGSV}\left\{(E_{M-H})^2\right\} \quad ...(S12)$$

$$S_{CH_3OH}^{CV-Mg} = c_{29}^{RGSV} + c_{30}^{RGSV}\left\{\frac{M_{cov}}{MC_{rad}}\right\} + c_{31}^{RGSV}\left\{(E_{M-H})^2\right\} \quad ...(S13)$$

S5. Details of the "ElemUp" model.

As shown in Figure S11, the lowest CV-RMSE of the "ElemUp" model is obtained at $Q = 1,\ D = 2$ (5.59%). The model with $Q = 1,\ D = 1$ shows almost comparable CV-RMSE (5.71%). Because the Training-RMSE of the $Q = 1,\ D = 2$ model is lower than that of the $Q = 1,\ D = 1$ model, $Q = 1,\ D = 2$ is adopted as the optimal complexity in the present study.

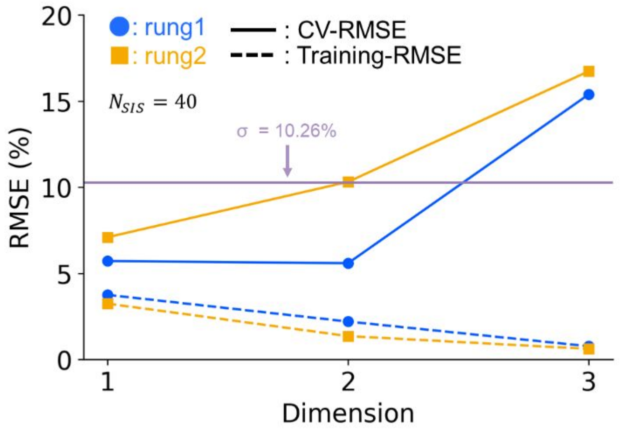

Figure S11. Training- and CV-RMSE curves of the "ElemUp" models. A purple horizontal line corresponds to the standard deviation of the CH_3OH selectivity of the new training data set including Co/V- and Zn-SBA-15 (σ = 10.26%).

Table S3. Coefficients of the "ElemUp" models.

Model	RGSV ($cm^3\ h^{-1}\ g_{cat}^{-1}$)	Coefficients		
		c_5^{RGSV}	c_6^{RGSV}	c_7^{RGSV}
ElemUp	2000	5.02E+00	1.00E-01	-1.33E+01
	4000	2.14E+01	8.35E-02	6.99E+00
	6000	3.47E+01	6.26E-02	2.47E+01
	8000	4.54E+01	3.68E-02	4.08E+01

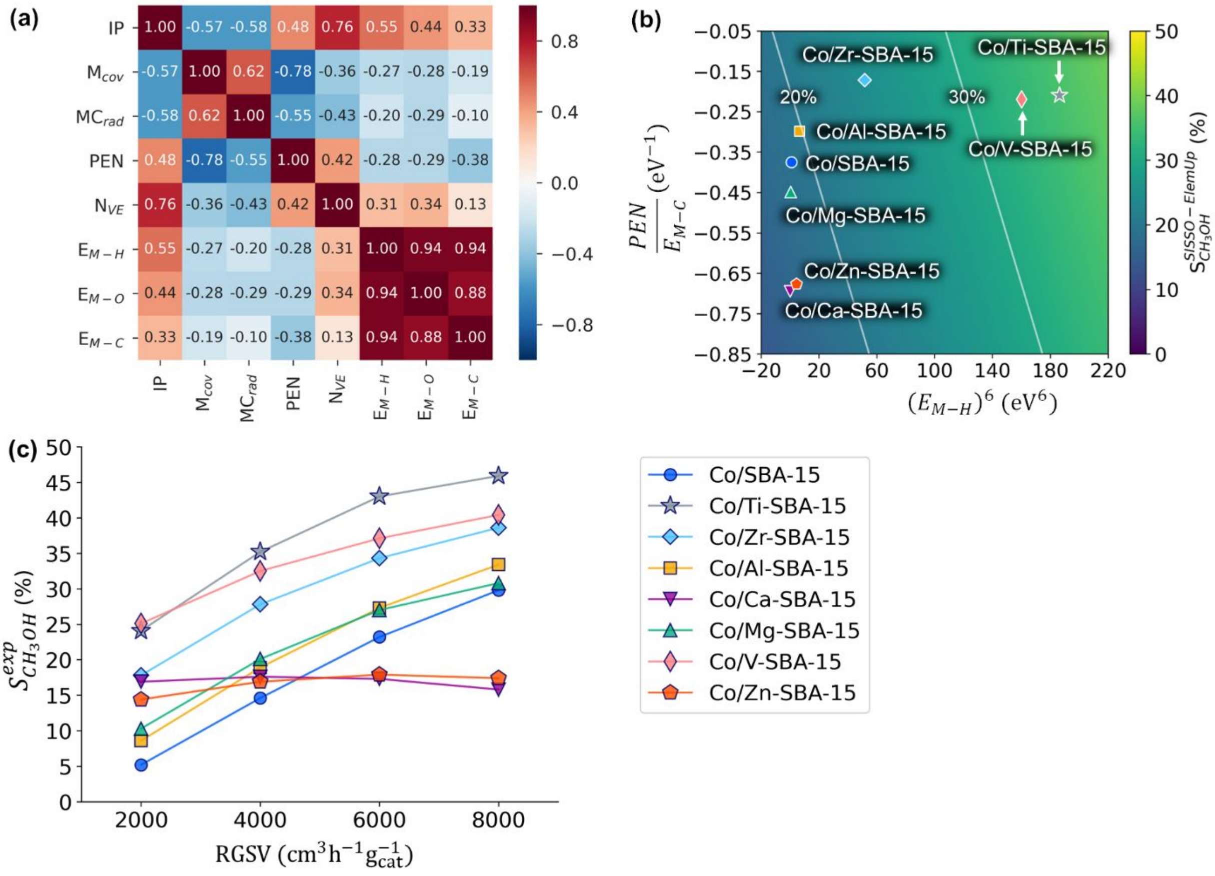

Figure S12. (a) The Pearson correlation matrix between the elemental features. (b) The catalyst map for the CH_3OH selectivity based on the "ElemUp" model. The x and y axis of the map are the descriptor components of the "ElemUp" model. (c) The experimental CH_3OH selectivity ($S_{CH_3OH}^{exp}$) along RGSV.

S6. The "Theo + Exp + Elem" model for the CH_4 and CO selectivity

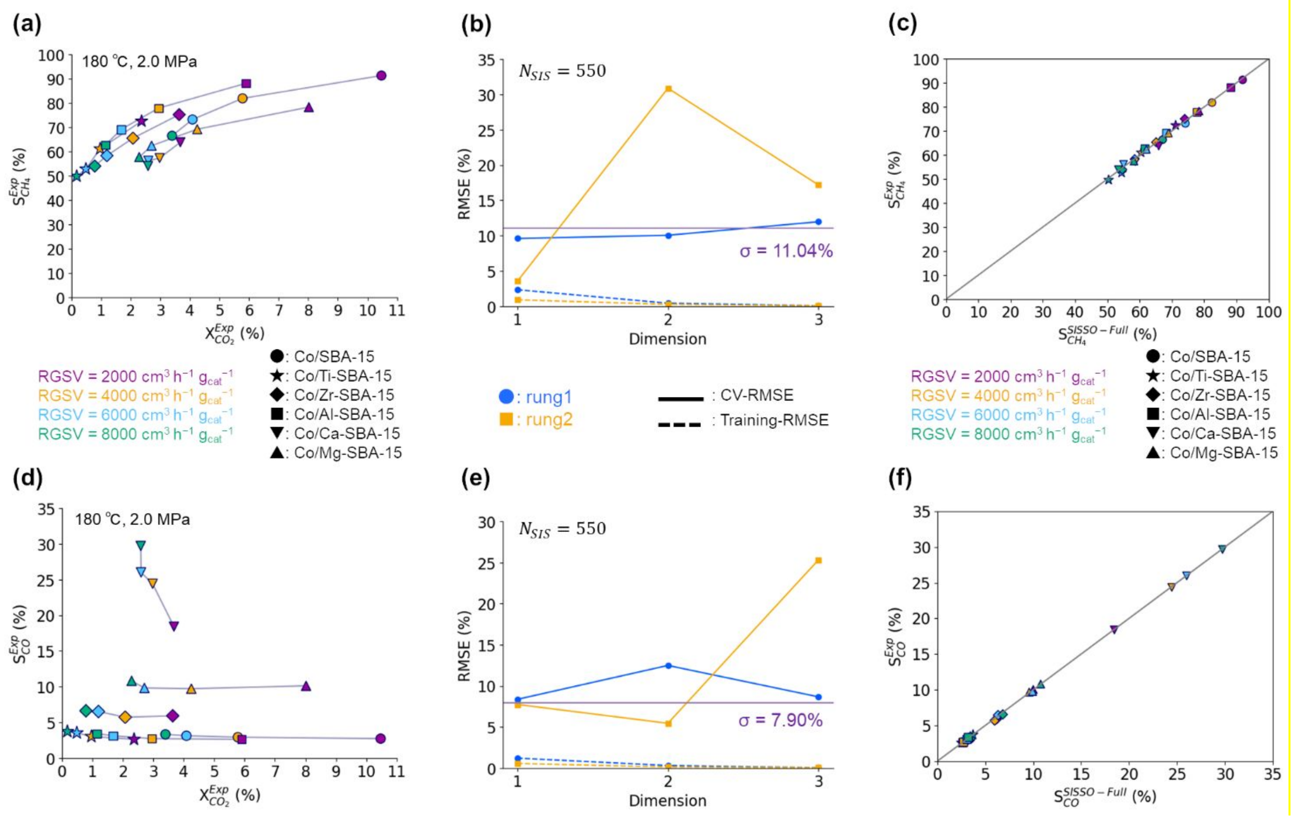

Figure S13. The experimental CH_4 selectivity ($S^{exp}_{CH_4}$) (a) and CO selectivity ($S^{exp}_{CH_4}$) (d) along the CO_2 conversion ($X^{RGSV}_{CO_2}$) of the Co/SBA-15 and Co/M-SBA-15 catalysts at different RGSV.[4] The 4 colors label the different RGSV and the 6 different shapes label the different catalysts. The lines connect RGSV values of the same catalysts. Reaction temperature and pressure are 180 ℃ and 2.0 MPa, respectively. Training- and CV-RMSE curves of the "Theo + Exp + Elem" models for the CH_4 selectivity (b) and the CO selectivity (e). A purple horizontal line corresponds to the standard deviation of the measured selectivity of the training data set (σ = 11.04% for CH_4 and σ = 7.90% for CO). Comparison between the experimental selectivity (y-axis) and the description provided by SISSO (x-axis) for the CH_4 selectivity (c) and the CO selectivity (f). The gray line shows the ideal relationship: $S^{exp} = S^{SISSO-Full}$.

Table S4. Coefficients of the "Theo + Exp + Elem" models for the CH_4 and CO selectivity.

Target	RGSV (cm^3 h^{-1} g_{cat}^{-1})	Coefficients		
		c_{32}^{RGSV}	c_{33}^{RGSV}	
CH_4 selectivity	2000	2.51E+01	5.37E+01	
	4000	3.00E+01	4.51E+01	
	6000	2.80E+01	4.30E+01	
	8000	2.25E+01	4.36E+01	
		c_{34}^{RGSV}	c_{35}^{RGSV}	c_{36}^{RGSV}
CO selectivity	2000	3.50E-01	1.25E-01	-1.72E+01
	4000	-2.69E+00	4.62E-02	-8.69E+00
	6000	-3.29E+00	3.05E-02	-6.59E+00
	8000	-4.15E+00	2.14E-02	-6.23E+00

S7. Experimental details

In the synthesis of V-SBA-15, ammonium metavanadate (NH_4VO_3) was used as the vanadium source.[10] Briefly, 4.0 g of the copolymer Pluronic P123 was dissolved in 120 mL of water by stirring for 4 h. An 0.49 g amount of NH_4VO_3 was added. After stirring for 1 h, an 8.67 g amount of tetraethyl orthosilicate (TEOS) was added dropwise. The pH value of the reaction mixture was adjusted to 2.0 by dropwise addition of 2 M HCl and the solution was stirred for 24 h at 40 °C. The suspension underwent hydrothermal treatment at 100 °C for 48 h under autogenous pressure. The resulting gel was filtered off, washed with water and absolute ethanol several times. After drying overnight at 80 °C, the final solid product was obtained from calcination in air at 550 °C for 12 h (1 °C min^{-1}).

The Zn-SBA-15 material was synthesized from a solution of 4.0 g of Pluronic P123 in 30 mL of

water and 120 mL of 2 M HCl by the addition of an 8.6 g amount of TEOS and 0.91 g of zinc acetate dihydrate ($ZnAc_2{\cdot}2H_2O$).[11-12] After stirring for 24 h at 40 °C, the suspension was kept in an oven at 100 °C for 48 h for hydrothermal treatment under autogenous pressure. The gel was dried by evaporation at 80 °C and in an oven overnight. Finally, the template was removed by calcination at 550 °C for 6 h (2 °C min^{-1}).

We have performed several experimental characterizations for Co/V- and Zn-SBA-15 catalysts. The bulk M/Si ratios of the Co/V- and Zn-SBA-15 materials (measured by SEM-EDX) are similar to the M/Si ratios of materials obtained previously in ref. 4 (Table S5). Thus, similar amount of V and Zn were incorporated into the SBA-15 supports of the new materials compared to the previously synthesized ones. Crucially, the SEM analysis (Figure S14) showed that V and Z are homogeneously distributed on the support. From the XRD patterns, Co/V- and Zn-SBA-15 show similar profiles corresponding to cobalt and cobalt oxide as those of Co/SBA-15 (Figure S15a), indicating the formation of Co nanoparticles. The N_2 adsorption isotherm of the Co/Zn-SBA-15 material is similar to that of the Co/SBA-15 material, whereas the isotherm of the Co/V-SBA-15 material shows a less well-defined ordered mesoporous structure compared to the Co/SBA-15 material (Figure S15b). In spite of the difference observed for the isotherm of the Co/V-SBA-15 material compared to Co/SBA-15, the surface areas, pore volumes, and pore diameters of the new materials, measured by N_2 physisorption, are comparable to those measured for the remaining materials (Table S5). This shows that the support has been retained during the incorporation of the V and Zn elements. Based on those characterizations, we conclude that the syntheses of Co/V- and Zn-SBA-15 were successful.

Table S5. Experimental characterization for Co/V- and Zn-SBA-15.

Catalyst	Bulk M/Si ratio	Specific surface area ($m^2\ g^{-1}$)	Total pore volume ($cm^3\ g^{-1}$)	Mean pore diameter (Å)
Co/V-SBA-15	0.100	558	1.31	90
Co/Zn-SBA-15	0.091	530	0.86	91
Co/SBA-15 [a]	0.000	665	0.77	77
Co/Ti-SBA-15 [a]	0.111	724	0.83	77
Co/Zr-SBA-15 [a]	0.100	664	0.91	91
Co/Al-SBA-15 [a]	0.071	518	0.92	119
Co/Ca-SBA-15 [a]	0.100	318	0.55	83
Co/Mg-SBA-15 [a]	0.083	501	0.69	84

[a]The results of Co/SBA-15 and Co/M-SBA-15 (M = Ti, Zr, Al, Ca, or Mg) were taken from our previous work.[4] Reproduced from [Belthle, K. S.; Beyazay, T.; Ochoa-Hernández, C.; Miyazaki, R.; Foppa, L.; Martin, W. F.; Tüysüz, H., Effects of Silica Modification (Mg, Al, Ca, Ti, and Zr) on Supported Cobalt Catalysts for H_2-Dependent CO_2 Reduction to Metabolic Intermediates. *J. Am. Chem. Soc.* **2022,** *144* (46), 21232-21243.]. Copyright 2022 American Chemical Society.

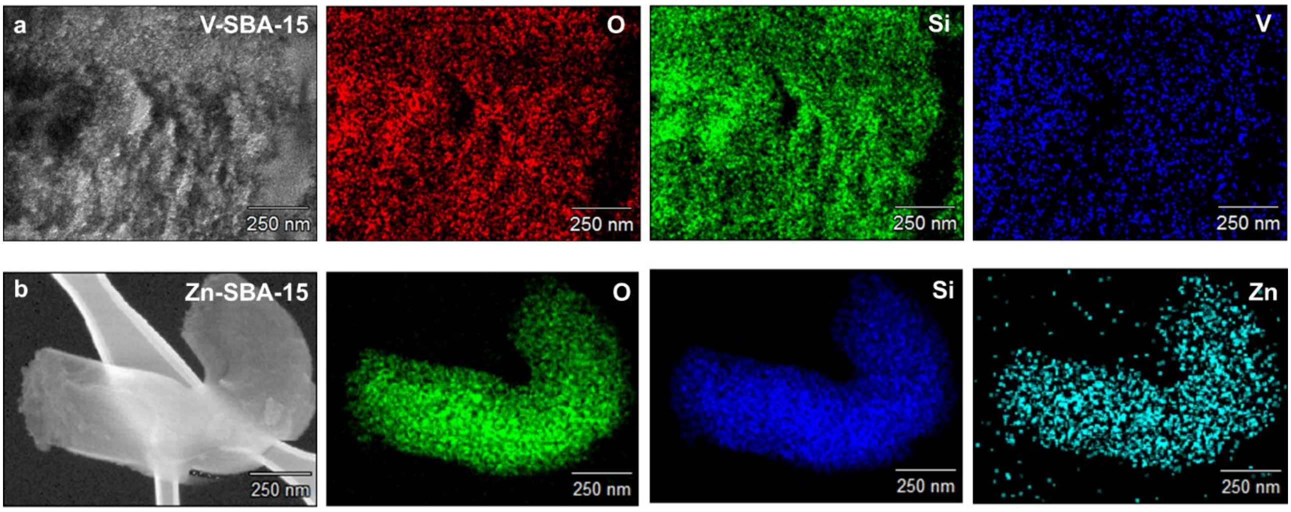

Figure S14. Dark-field HR-STEM micrographs and SEM-EDX elemental mappings of a) V-SBA-15 and b) Zn-SBA-15 support materials.

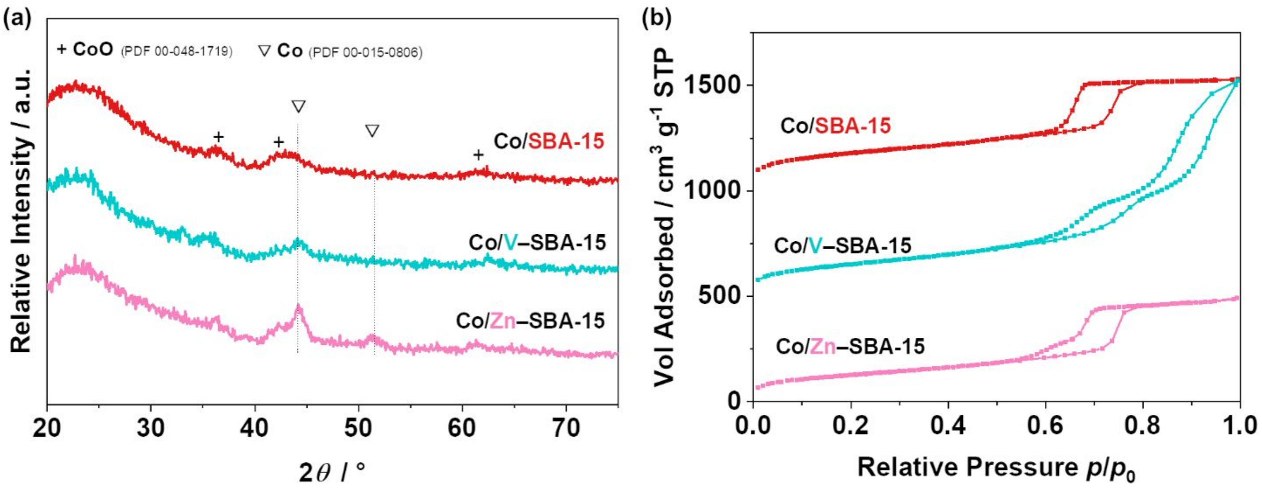

Figure S15. (a) Wide-angle XRD patterns and (b) N_2 adsorption isotherms of Co/SBA-15, Co/ V-SBA-15, and Co/ Zn-SBA-15. The isotherms of Co/ V-SBA-15 and Co/ Zn-SBA-15 are shifted by 500 cm^3/g for better clarity.

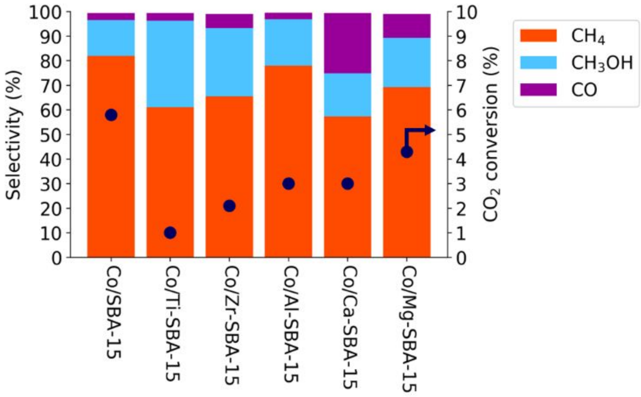

Figure S16. Catalytic performance of the Co/SBA-15 and Co/M-SBA-15 catalysts.[4] Color bars and black dots show the selectivity of gas-phase organic molecules and CO_2 conversion of the different catalysts, respectively. Reproduced from [Belthle, K. S.; Beyazay, T.; Ochoa-Hernández, C.; Miyazaki, R.; Foppa, L.; Martin, W. F.; Tüysüz, H., Effects of Silica Modification (Mg, Al, Ca, Ti, and Zr) on Supported Cobalt Catalysts for H_2-Dependent CO_2 Reduction to Metabolic Intermediates. *J. Am. Chem. Soc.* **2022,** *144* (46), 21232-21243.]. Copyright 2022 American Chemical Society.

REFERENCES

1. Purcell, T. A. R.; Scheffler, M.; Ghiringhelli, L. M., Recent advances in the SISSO method and their implementation in the SISSO++ code. **2023**, arXiv:2305.01242v1.

2. Purcell, T. A. R.; Scheffler, M.; Carbogno, C.; Ghiringhelli, L. M., SISSO++: A C++ Implementation of the Sure-Independence Screening and Sparsifying Operator Approach. *J. Open Source Softw.* **2022,** *7* (71), 3960.

3. Comas-Vives, A., Amorphous SiO_2 surface models: energetics of the dehydroxylation process, strain, ab initio atomistic thermodynamics and IR spectroscopic signatures. *Phys. Chem. Chem. Phys.* **2016,** *18* (10), 7475-7482.

4. Belthle, K. S.; Beyazay, T.; Ochoa-Hernández, C.; Miyazaki, R.; Foppa, L.; Martin, W. F.; Tüysüz, H., Effects of Silica Modification (Mg, Al, Ca, Ti, and Zr) on Supported Cobalt Catalysts for H_2-Dependent CO_2 Reduction to Metabolic Intermediates. *J. Am. Chem. Soc.* **2022,** *144* (46), 21232-21243.

5. Farkaš, B.; Terranova, U.; de Leeuw, N. H., Binding modes of carboxylic acids on cobalt nanoparticles. *Phys. Chem. Chem. Phys.* **2020,** *22* (3), 985-996.

6. Hammer, B.; Hansen, L. B.; Nørskov, J. K., Improved adsorption energetics within density-functional theory using revised Perdew-Burke-Ernzerhof functionals. *Phys. Rev. B* **1999,** *59* (11), 7413-7421.

7. Blum, V.; Gehrke, R.; Hanke, F.; Havu, P.; Havu, V.; Ren, X.; Reuter, K.; Scheffler, M., Ab initio molecular simulations with numeric atom-centered orbitals. *Comput. Phys. Commun.* **2009,** *180* (11), 2175-2196.

8. Weststrate, C. J.; van de Loosdrecht, J.; Niemantsverdriet, J. W., Spectroscopic insights into cobalt-catalyzed Fischer-Tropsch synthesis: A review of the carbon monoxide interaction with single crystalline surfaces of cobalt. *J. Catal.* **2016,** *342*, 1-16.

9. Adamo, C.; Barone, V., Toward reliable density functional methods without adjustable parameters: The PBE0 model. *J. Chem. Phys.* **1999,** *110* (13), 6158-6170.

10. Kunkel, B.; Kabelitz, A.; Buzanich, A. G.; Wohlrab, S., Increasing the Efficiency of Optimized V-SBA-15 Catalysts in the Selective Oxidation of Methane to Formaldehyde by Artificial Neural Network Modelling. *Catalysts* **2020,** *10* (12), 1411.

11. Liu, M.; Gao, K.; Liang, L.; Sun, J.; Sheng, L.; Arai, M., Experimental and theoretical insights into binary Zn-SBA-15/KI catalysts for the selective coupling of CO_2 and epoxides into cyclic carbonates under mild conditions. *Catal. Sci. Technol.* **2016,** *6* (16), 6406-6416.

12. Zhang, J.; Cheng, K.; Li, H.; Yin, F.; Wang, Q.; Cui, L.; Yang, S.; Nie, J.; Zhou, D.; Zhu, B., Efficient Synthesis of Structured Phospholipids Containing Short-Chain Fatty Acids over a Sulfonated Zn-SBA-15 Catalyst. *J. Agric. Food. Chem.* **2020,** *68* (44), 12444-12453.
